# Analysis of the anti-Alzheimer potential of bioactive compounds from *Citrus hystrix* DC. peel, leaf, and essential oil by network pharmacology

**DOI:** 10.1016/j.heliyon.2024.e33496

**Published:** 2024-06-26

**Authors:** Adhisa Fathirisari Putri, Didik Huswo Utomo, Woro Anindito Sri Tunjung, Wahyu Aristyaning Putri

**Affiliations:** a Faculty of Biology, Universitas Gadjah Mada, Jl. Teknika Selatan, Sekip Utara, Yogyakarta, 55281, Indonesia; b Bioinformatics Research Center, INBIO-Indonesia, Malang, 65162, Indonesia; c Biosystem Education Center, Brawijaya University, Malang, 65145, Indonesia

**Keywords:** Alzheimer's disease, *Citrus hystrix*, protein network, network pharmacology, molecular docking, molecular dynamics

## Abstract

Alzheimer's disease (AD) is the most known neurodegenerative disease, and its prevalence is predicted to increase significantly. Discovering novel drugs and treatments for AD is urgently needed. Drugs from natural products have been preferred lately due to their high potential and low toxicity. *Citrus hystrix* DC. (kaffir lime; KL) is one such herbal plant that is found abundantly in Southeast Asia with many biological activities. In this study, the potential of bioactive compounds from KL peel, leaf, and essential oil as anti-AD agents was explored using network pharmacology. First, the compounds were identified with KNApSAcK database and related literature. Subsequently, the targets of each corresponding compound were determined with SEA Search Server and Swiss Target Prediction, while the proteins associated with AD were identified using OMIM, GenCLiP3, and DisGeNET. Furthermore, a protein–protein interaction network and a compound–target interaction network were constructed to identify the most crucial proteins and compounds in the network by employing Cytoscape v3.9.1. The study continued with pathway enrichment analysis using STRING v1.7.1, molecular docking with PyRx and SwissDock, and molecular dynamics simulation with YASARA for further confirmation. Our results showed that almost all the secondary metabolites of KL targeted AD-associated genes, with oxypeucedanin and citrusoside A showing the highest anti-AD potential and targeting essential genes, EGFR and MAPK14, respectively. These targets were associated with inflammatory and oxidative stress pathways, indicating the potential mechanism of KL in attenuating AD clinical manifestation.

## 1 Introduction

Alzheimer's disease (AD) is a progressive neurodegenerative disease with a strong correlation with age. One of the earliest symptoms of this disease is the impairment of cognitive functions, which may lead to the weakening of other vital body functions and even death. The prevalence of AD is estimated to increase rapidly in the middle of the century, affecting approximately 13,8 million Americans aged >65 years old [1].

AD is a multifactorial disease that could be characterized by various cellular and molecular processes; although the actual cause of this disease remains unknown, age is believed to be the highest risk factor [[2], [3], [4], [5], [6], [7]]. Studies have shown a significant correlation between aging and numerous AD pathogenesis [[10], [11], [8], [9]]. Several hypotheses have been proposed to explain the cause and mechanism of AD, such as the accumulation of β-amyloid (Aβ) plaques and neurofibrillary tangles in neurons, cholinergic neurons, neuroinflammation, and oxidative activity in the brain [3,5,[12], [13], [14], [15]]. Such factors cause synaptic failure and neuronal death, leading to brain atrophy and decreased brain functions [3,5,[12], [13], [14], [15], [16]].

Till date, no pharmacological treatment has been developed that can prevent or stop biological activities promoting AD progression. Current available treatments only address symptoms and may have mild side effects [2,3,[13], [14], [15]]. Hence, an effective anti-AD is urgently needed.

Treatment with natural products is often preferred due to its multitarget mechanism which enables it to target different factors that promote disease progression [[17], [18], [19], [20]]. *Citrus hystrix* DC. (kaffir lime; KL) is a citrus plant that is abundant in Southeast Asia, including Indonesia. It has several beneficial parts, such as leaf, fruit, peel, and essential oil, which are rich in bioactive compounds. Previous studies have shown that KL may have some potential in managing AD. In an in vitro study using a neuronal senescent model on SH-SY5Y cells, Pattarachotanat and Tencomnao (2020) [21] reported that the leaf and peel extracts of KL exhibit neuroprotective ability against senescence induced by high blood glucose levels and might have potential as an anti-AD agent.

In addition to its leaf and peel, KL essential oil is used as an aromatherapy ingredient. Aromatherapy has been proposed as an AD treatment [[22], [23], [24], [25]], possibly owing to its capability to improve cognitive functions through olfactory pathways [26]. Moreover, bioactive compounds of KL, such as sitosterol, are known to exhibit anti-AD properties. Sitoserol is reported to be a good inhibitor of cholinesterase (AChE), capable of preventing glutamate and Aβ toxicity, and possesses antioxidant activity [21,[27], [28], [29]].

However, the potential and mechanism of KL in managing AD are yet to be explored. For elucidating such multitarget mechanism, network pharmacology can be utilized to understand the system and process of how KL may cure AD through various pathologic pathways. The network pharmacology approach aims to understand how molecules act on a system level, impacting multiple targets and pathways simultaneously rather than concentrating on just one. It involves analyzing biological networks to gain insight into the molecular mechanisms of drugs and diseases [30,31]. This method shows promise for drug discovery, particularly in complex, multifactorial diseases like AD.

This study attempts to explore the potential mechanism of KL in attenuating AD by applying network pharmacology to its bioactive compounds against AD targets. In addition, molecular docking and molecular dynamics simulations are performed to validate the network analysis results. Our findings indicated that oxypeucedanin and citrusoside A have potential as inhibitors of EGFR and MAPK14, which could potentially improve symptoms of AD by suppressing related inflammatory and oxidative stress pathways.

## 2 Materials and methods

### 2.1 Identification of bioactive compounds

The bioactive compounds of KL leaf, peel, and essential oil (from blossom, leaf, branch, and fruit peel) were identified from the KNApSAck database (http://www.knapsackfamily.com/KNApSAcK/) [32], Dr. Duke database (https://phytochem.nal.usda.gov/phytochem/search), and previous reports [21,33]. A total of 69 compounds were combined and deduplicated, and the secondary metabolites were selected. Finally, 52 secondary metabolites were included in the analysis.

### 2.2 Identification of compound-targeted genes and AD-associated genes

The SMILES of each identified secondary metabolite was collected from PubChem database (https://pubchem.ncbi.nlm.nih.gov/) [34] and used for target identification using SEA Search Server (https://sea.bkslab.org/) [35] and Swiss Target Prediction (STP) (http://www.swisstargetprediction.ch/) [36] with the *Homo sapiens* filter. The top three targets with the highest scores were selected from each database. The top 10 genes in cases where all the identified genes had the same score were chosen. All the results were combined and deduplicated. AD-associated genes were also collected from DisGeNET database (https://www.disgenet.org/) [37], Online Mendelian Inheritance in Man (OMIM) (https://omim.org/) [38], and GenCLiP3 database (http://ci.smu.edu.cn/genclip3/analysis.php) [39] with the *Homo sapiens* filter, combined, and deduplicated. Both lists of genes were entered to Venny 2.1. to find the overlapping genes, generating 64 KL target genes that were also AD-associated genes.

### 2.3 Construction of a Protein–Protein Interaction (PPI) network

The overlapping genes were inserted to STRING v1.7.1. integrated with Cytoscape v3.9.1 [40]. as organism *Homo sapiens* to visualize the interaction of proteins. The top 10 most essential genes in the network were identified using another application integrated with Cytoscape, CytoHubba v0.1 [41] through the maximal clique centrality (MCC) algorithm. The redder the color of the node, the higher its MCC scores.

### 2.4 Construction of a Compound–Target Interaction (CTI) network

A CTI network was constructed manually from the target identification results using Cytoscape v3.9.1. to map the interaction between protein targets and their corresponding targeting compounds. The target nodes were colored as yellow to red according to their MCC score, and the compound nodes were yellow. The edges show the association between the compound and its targets.

### 2.5 Analysis of KEGG pathway enrichment

The previously constructed PPI network was further analyzed by Kyoto Encyclopedia of Genes and Genomes (KEGG) pathway enrichment using STRING Enrichment App integrated with Cytoscape. When retrieving the functional enrichment, the background network was set to be the genome, providing a list of pathways along with their corresponding genes, number of background genes, P-value, and false discovery rate (FDR; Q) value. The pathways were selected and filtered with Q-value <0.001. Additionally, the extent of pathway enrichment was determined by computing the rich factor, which was obtained by dividing the number of genes in a specific pathway within the network by the number of background genes. Subsequently, data from these 14 highly enriched pathways was consolidated and presented visually using Datylon (https://www.datylon.com).

### 2.6 Preparation of proteins and ligands

Molecular docking was conducted to validate the results of the network analysis. Epidermal growth factor receptor (EGFR) (PDB ID: 7B85; deposited by [42], [43]), mitogen-activated protein kinase 14 (MAPK14) (PDB ID: 6HWU; deposited by [44], [45]), and peroxisome proliferator activated receptor alpha (PPARA) (PDB ID: 6KXY; deposited by [46], [47]) were selected as the proteins. The selection was made based on the results of target identification, MCC scores, and involvement in the most enriched pathway. Their 3D structures were searched in the Uniprot (https://www.uniprot.org) [48] and RCSB PDB database (https://www.rcsb.org/). The protein's crystal structure was chosen based on various factors including the role of the native ligand bound to the protein, methodology, resolution, completeness of the protein chain or domain, deposition dates, and presence of any absent residues or mutations. The structural integrity was additionally validated using Ramachandran Plot statistics by PROCHECK accessed through PDBsum (https://www.ebi.ac.uk/thornton-srv/databases/pdbsum/), with only structures exhibiting a value exceeding 90 % being ultimately selected for docking. The selected structures were then prepared with PyMOL v2.0 [49] by removing water and nonstandard (ligand) molecules. If any missing residues were found, the structure was repaired using Dock Prep in Chimera v1.1.6 [50].

The bioactive compounds targeting these three proteins based on the previous identification target results were selected as the ligands, and the 3D structures of these bioactive compounds were obtained from PubChem as.sdf files. On the other hand, the controls for each protein were their respective native ligands obtained from the RCSB database. The chosen controls were propan-2-yl 2-[[4-[2-(dimethylamino)ethyl-methyl-amino]-2-methoxy-5-(propanoylamino)phenyl]amino]-4-(1-methylindol-3-yl)pyrimidine-5-carboxylate (R28) for EGFR, 3-(2,5-dimethoxyphenyl)-∼{*N*}-[4-[4-(4-fluorophenyl)-2-[(∼{E})-phenyldiazenyl]-1,3-thiazol-5-yl]pyridin-2-yl]propanamide (GE5) for MAPK14, and 6-ethyl-1-(4-fluorophenyl)-3-pentan-3-yl-pyrazolo[3,4-*b*]pyridine-4-carboxylic acid (T06) for PPARA.

The protein and ligand conformations were additionally prepared using PyRx and SwissDock, correspondingly. In the case of PyRx, the ligand structures were prepared in OpenBabel integrated with PyRx v0.9.8 [51]. by minimizing the energy with the Universal Force Field (UFF) using the conjugate gradient algorithm. The total number of steps was defined as 200 and the number of steps for update was set to 1. In addition, the procedure was configured to stop if the energy difference dropped below 0.1 kcal/mol. After performing ligand energy minimization, the proteins and compounds were inserted into the AutoDock Wizard integrated with PyRx. This involved designating them as macromolecules and ligands respectively. This step automatically added hydrogen molecules, merged non-polar hydrogens, and added Gasteiger charges to the structures, converting them into.pdbqt files ready for docking.

In the case of SwissDock (https://www.swissdock.ch/) [52], Attracting Cavities 2.0 [53] engine was chosen for docking. Ligand data was transformed into Mol2 files through PyMOL v2.0 and then uploaded onto the platform for processing. SwissParam with the MMFF-based method was utilized for parameterization. PDB IDs were provided for the targets, selecting the A chain in cases where multiple chains existed, and specifying no heteroatom. The preparation of targets was carried out to facilitate their utilization in CHARMM simulations.

### 2.7 Molecular docking: AutoDock Vina (PyRx)

Targeted molecular docking was performed for each protein in AutoDock Vina integrated with PyRx v0.9.8. The exhaustiveness and the mode were set to 50 and 9, respectively. Prior to the actual docking using the selected ligands, a validation was conducted by redocking the proteins with each corresponding native ligand (control). The redocking process was carried out repeatedly until the RMSD (Root Mean Square Deviation) result was less than 2 Å, demonstrating its conformational similarity with the co-crystallized control and confirming the accuracy of the docking procedure [[53], [54], [55], [56]]. The RMSD values were calculated using DockRMSD (https://zhanggroup.org/DockRMSD/) [57].

Afterward, molecular docking was performed with the prepared proteins and ligands using identical grid box coordinates employed for redocking as shown in Table S5. Subsequently, the best docking poses were combined with their respective protein structures utilizing PyMOL v2.5.2. These complexes were further analyzed and visualized with Discovery Studio v21.1.0.20298 [58] to determine the binding types and residues involved in the interactions.

### 2.8 Molecular docking: Attracting Cavities 2.0 (SwissDock)

The same proteins and ligands were also subjected to molecular docking using another software known as Attracting Cavities 2.0, which is accessible through the SwissDock web-based server. Once the ligand and target were prepared, a search space was defined using the identical grid box coordinates employed for docking with Vina. Additionally, parameters were configured based on default settings, such as setting the number of RIC to 1, choosing medium for sampling exhaustivity, and choosing buried for cavity prioritization. The scoring function comprises the AC score, which considers CHARMM force field energy and fast analytical continuum treatment of solvation (FACTS) terms, as well as the SwissParam score, a weighted sum of polar and nonpolar terms. The AC score was employed for selecting the optimal model, whereas the SwissParam score was computed to assess the affinity of various compounds towards a specific target. The docking outcomes were accessed using ViewDock in Chimera v1.16, and the most favorable samples were stored as.pdb files for further examination in Discovery Studio v21.1.0.20298.

### 2.9 Molecular dynamics simulation

The top docked complexes, EGFR-oxypeucedanin and MAPK14-citrusoside A, were further investigated by performing molecular dynamics simulation in YASARA v21.12.19 [59] for a period of 10.00 ns with a timestep of 2.5 fs. The md_runfast.mcr script was used to define parameters such as the AMBER14 force field, temperature of 310K, pH of 7.4, NaCl concentration of 0.9 %, and pressure at 1 atm. The MD simulation was also performed on the control complexes for comparison purposes. The findings were assessed through the execution of the md_analyze.mcr script to gain insights into the RMSD Cα, RMSD ligand conformation, and RMSD ligand movement.

## 3 Results

### 3.1 Identification of compound-targeted genes and AD-associated genes

The KL compounds were gathered from KNApSAck, Dr. Duke, and a few research papers [21,33]. The KNApSAck database yielded 45 compounds from volatiles of blossom extract [60] and fruit peel extract [61]. In contrast, the Dr. Duke database did not yield any results. Warsito et al. (2017) [33] reported 28 compounds derived from KL leaf, branch, and fruit peel essential oils. Finally, Pattarachotanat & Tencomnao (2020) [21] documented a total of 27 compounds combined from the leaf and fruit peel extracts. These compounds were combined and deduplicated, resulting in a total of 69 compounds as displayed in Table S1. From this pool of compounds, 52 secondary metabolites were derived due to their diverse pharmacological activities.

**Fig. 1:**
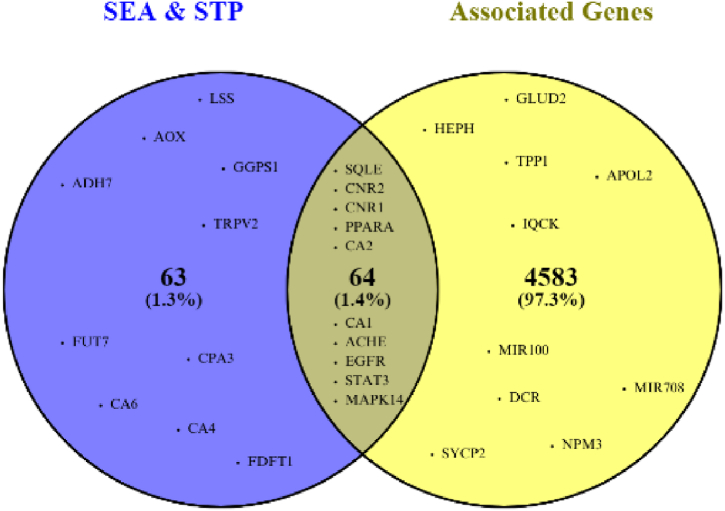
Venn diagram of compound-targeted genes (collected from SEA Search Server and Swiss Target Prediction) and Alzheimer-associated genes (collected from DisGeNET, OMIM, and GenCLiP3).

The targets of each compound were then identified using SEA Search Server and STP, which generated 127 target genes (Table S2). AD-associated genes were collected from three databases (DisGeNET, OMIM, and GenCLiP3), resulting in 4647 genes after deduplication. A Venn diagram was created using Venny 2.1. to find the intersection of both lists of genes, resulting in 64 KL-targeted genes that were also associated with AD (Fig. 1.).

### 3.2 Protein–Protein Interaction (PPI) network

**Fig. 2:**
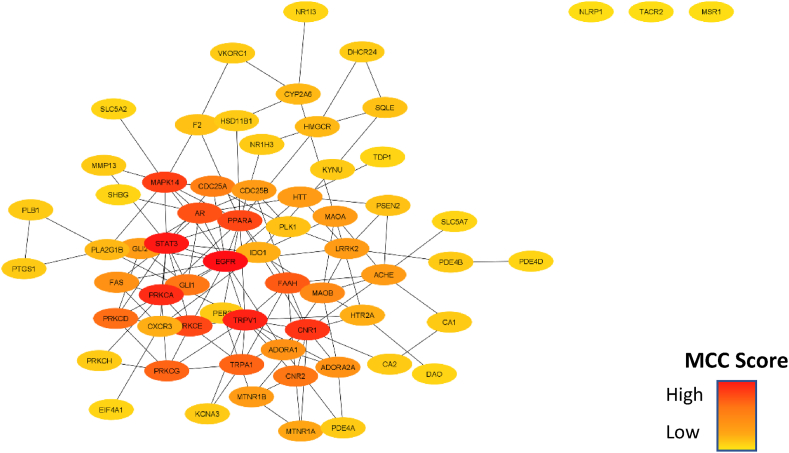
PPI network constructed with Cytoscape v3.9.1. Each node represents the overlapping proteins from the previous step, and the edges show the interaction of the nodes. The thick red color of the node reveals the node's importance in the network based on its MCC score. (For interpretation of the references to color in this figure legend, the reader is referred to the Web version of this article.)

The 64 intersecting genes were inputted to STRING v1.7.1. integrated with Cytoscape v3.9.1. to construct the PPI network. The network consisted of 64 nodes, 135 edges, and 4.426 average neighbors. All proteins had interactions with each other except for NLRP1, TACR2, and MSR1. The MCC algorithm in CytoHubba v0.1., which is also integrated with Cytoscape, was then used to find the most crucial genes in the network. The redder the color of the node, the higher the MCC score and the greater the importance of the node in the network. The top 10 most essential genes were identified based on MCC as follows: EGFR, STAT3, TRPV1, PRKCA, CNR1, MAPK14, PPARA, PRKCE, AR, and FAAH (Fig. 2.). The exact scores are listed in Table S3.

### 3.3 Compound–Target Interaction (CTI) network

**Fig. 3:**
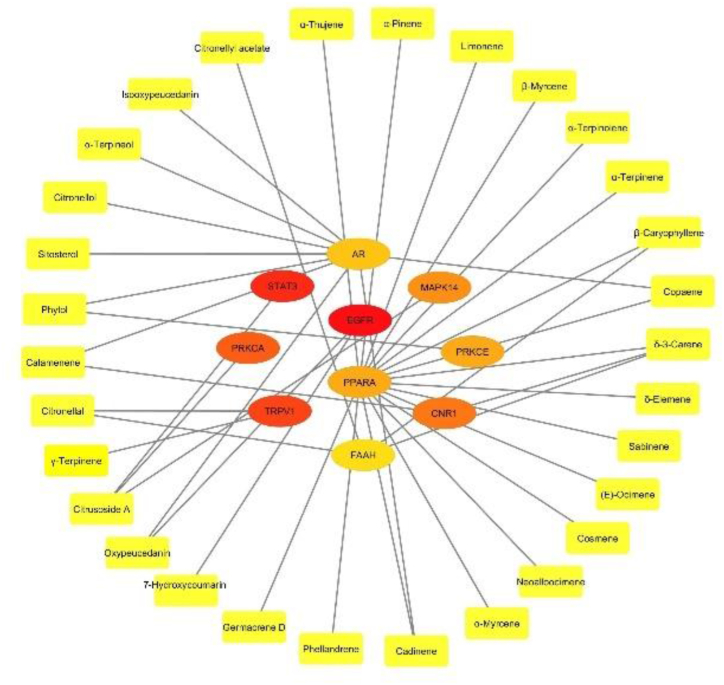
Compound–target interaction network based on target identification results of the top 10 most essential genes of the previous PPI network constructed with Cytoscape v3.9.1. The genes are represented with yellow-red circular nodes, and the compounds are shown as yellow rectangular nodes. The edges reveal the interaction between each compound and its targets. (For interpretation of the references to color in this figure legend, the reader is referred to the Web version of this article.)

The top 10 most essential genes were further used to analyze their interactions with the corresponding KL compounds on the basis of the previous target identification results in Fig. 3. The network was constructed using Cytoscape v3.9.1. with yellow-red circular nodes as genes, yellow rectangular nodes as compounds, and edges as compound–target interactions. The results revealed 30 KL compounds targeting the top 10 genes. This value comprised more than half of the total secondary metabolites, revealing the multitarget capability of KL. Among the compounds, δ-3-carene and citrusoside A had the most interactions with targets. Among the genes, PPARA was the most targeted by the KL compounds.

A full CTI network showing the interaction between all targets and the corresponding compounds revealed that all KL secondary metabolites targeted the AD-associated genes except α-bergamotene. Oxypeucedanin and phytol had the most prevalent interaction with the targets (Fig. S1).

### 3.4 KEGG pathway enrichment analysis

Fourteen signaling pathways were obtained from the KEGG enrichment analysis of the previous PPI network filtered with false discovery rate (FDR) < 0.001 to include only the statistically significant pathways. FDR value serves as a synonym for Q-value and represents the corrected P-value based on the Benjamini—Hochberg method [62]. Additionally, these pathways exhibited high rich factors and high gene counts, underscoring their significance in the network (Fig. S2). This finding demonstrated the possible mechanism of KL secondary metabolites in modulating AD via various pathways such as inflammation and oxidative stress through TRP channels, parathyroid hormone, and metabolic pathways. Among these pathways, the inflammatory mediator regulation of TRP channels was the most enriched with a high rich factor, a low Q-value, and a high gene count. The details of all pathways are listed in Table S3.

### 3.5 Molecular docking

To validate the network analysis results, molecular docking was performed with EGFR, MAPK14, and PPARA as the proteins and their corresponding compounds as the ligands. These proteins were chosen because of their high MCC scores and involvement in neuroinflammation and oxidative pathways leading to AD, the most enriched pathways based on the previous result.

Prior to the actual docking procedures, the control ligands were redocked to its corresponding protein to validate the accuracy of the docking procedure. The RMSD results from this exercise were consistently under 2 Å for all controls, as detailed in Table 1. This confirmed the precision of the docking simulation, and consequently, the grid box measurements of the correct binding site were employed for the actual experiment.

**Table 1:** RMSD (Å) of the redocked native ligands to validate the accuracy of the docking procedure.

No	Protein	Native Ligand	RMSD (Å)
**1**	EGFR (7B85)	R28	1.308
**2**	MAPK14 (6HWU)	GE5	1.588
**3**	PPARA (6KXY)	T06	0.785

Docking was initially performed using AutoDock Vina in PyRx v0.9.8. and the binding affinity outcomes are presented in Table 2. Wong et al. (2022) [63] have categorized complexes with a binding energy of < −7 kcal/mol as indicative of strong predicted binding, < −5 kcal/mol as moderate predicted binding, and > −5 kcal/mol as no predicted binding. Based on this classification, the majority of the docked complexes exhibited moderate binding tendencies. Furthermore, the results showed that no compound had better binding affinity than the controls. Nevertheless, several compounds exhibited strong predicted binding with similar values to the controls, such as oxypeucedanin (EGFR), citrusoside A (MAPK14), β-caryophyllene (PPARA), germacrene D (PPARA), and copaene (PPARA). Additionally, δ-elemene (PPARA) also demonstrated a relatively good binding affinity (Table 1).

**Table 2:** Binding affinity (kcal/mol) from the molecular docking results of each docked complex using AutoDock Vina and Attracting Cavities 2.0. Complexes with the best binding affinities (strong predicted binding) are highlighted in bold.

No	Protein	Compound	AutoDock Vina Binding Affinity (kcal/mol)	SwissParam Score (kcal/mol)
**1**	EGFR (7B85)	R28 (Control)	−9	−9.1
		7-Hydroxycoumarin	−6	−6.5
		**Oxypeucedanin**	**−7.9**	**−7.6**
**2**	MAPK14 (6HWU)	GE5 (Control)	−8.7	−10.3
		**Citrusoside A**	**−7.7**	**−8.1**
**3**	PPARA (6KXY)	T06 (Control)	−9.4	−8.6
		α-Thujene	−5.9	−6.4
		α-Pinene	−5.7	−6.5
		Limonene	−5.9	−6.5
		β-Myrcene	−5.3	−6.6
		α-Terpinolene	−6.3	−6.4
		α-Terpinene	−6	−6.5
		**β-Caryophyllene**	**−8.2**	−6.0
		**Copaene**	**−7.5**	**−7.1**
		δ-3-Carene	−6.2	−6.4
		**δ-Elemene**	−6.8	**−7.1**
		Sabinene	−6	−6.5
		(*E*)-Ocimene	−5.9	−6.6
		Cosmene	−5.8	−6.6
		Neoalloocimene	−6.2	−6.6
		α-Myrcene	−5.2	−6.6
		Cadinene	−6.6	−6.4
		Phellandrene	−5.9	−6.5
		**Germacrene D**	**−7.8**	**−7.0**

The docking experiment was also conducted with another software known as AC in SwissDock. This procedure yielded two sets of scores based on AC and SwissParam scoring functions. The AC scores can be found in the supplementary section (Table S6), while the SwissParam scores are featured in Table 2, reflecting the binding energy between the ligand and protein. The scores fell within a similar range as the scores obtained from Vina docking, validating the docking outcomes. While no compound exhibited superior binding affinity compared to the controls, certain compounds such as oxypeucedanin (EGFR), citrusoside A (MAPK14), copaene (PPARA), and δ-elemene (PPARA) still demonstrated strong predicted binding.

**Fig. 4:**
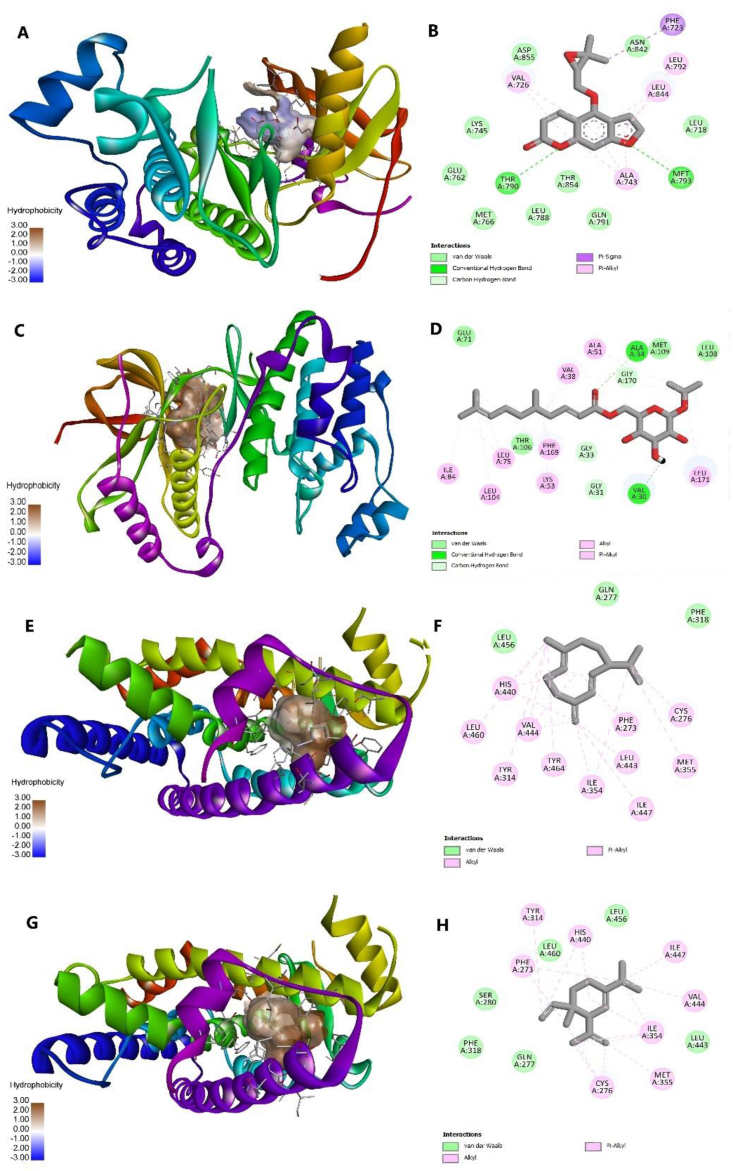
AutoDock Vina docking visualization of EGFR–oxypeucedanin (A–B), MAPK–citrusoside A (C–D), PPARA–germacrene D (E–F), and PPARA–δ-elemene (G–H) using Discovery Studio. The visualization on the left shows the protein structure and its hydrophobicity, and the right picture reveals the residue interaction of the complex.

The Vina docking result was further analyzed using Discovery Studio to determine the interacting residues and bonds formed between protein and ligand as displayed in Fig. 4. EGFR–oxypeucedanin complex had relatively low hydrophobicity as shown by the white-to-blue color, but the complex retained some of the main interacting residues in control (R28) including the hydrogen bonds, namely, THR790 and MET793. MAPK14–citrusoside A complex had good hydrophobicity as revealed by the brown color, preserved the interacting residues in control (GE5), and had a sufficient number of hydrogen bonds. Similar interactions were observed with GLY170 serving as the residue for hydrogen bonds, akin to the control. Additionally, other residues including ALA34, VAL30, GLY31, and GLY33 were also involved in the hydrogen bonding. Subsequently, PPARA complexes with germacrene D and δ-elemene also showed good hydrophobicity and maintained some of the interacting residues. However, PPARA complexes had no hydrogen bonds and thus were expected to be unstable. For the other PPARA corresponding compounds, they exhibited low affinities and low similarities with the control in terms of interacting residues. Complete information on residue interactions can be found in Table S7.

**Fig. 5:**
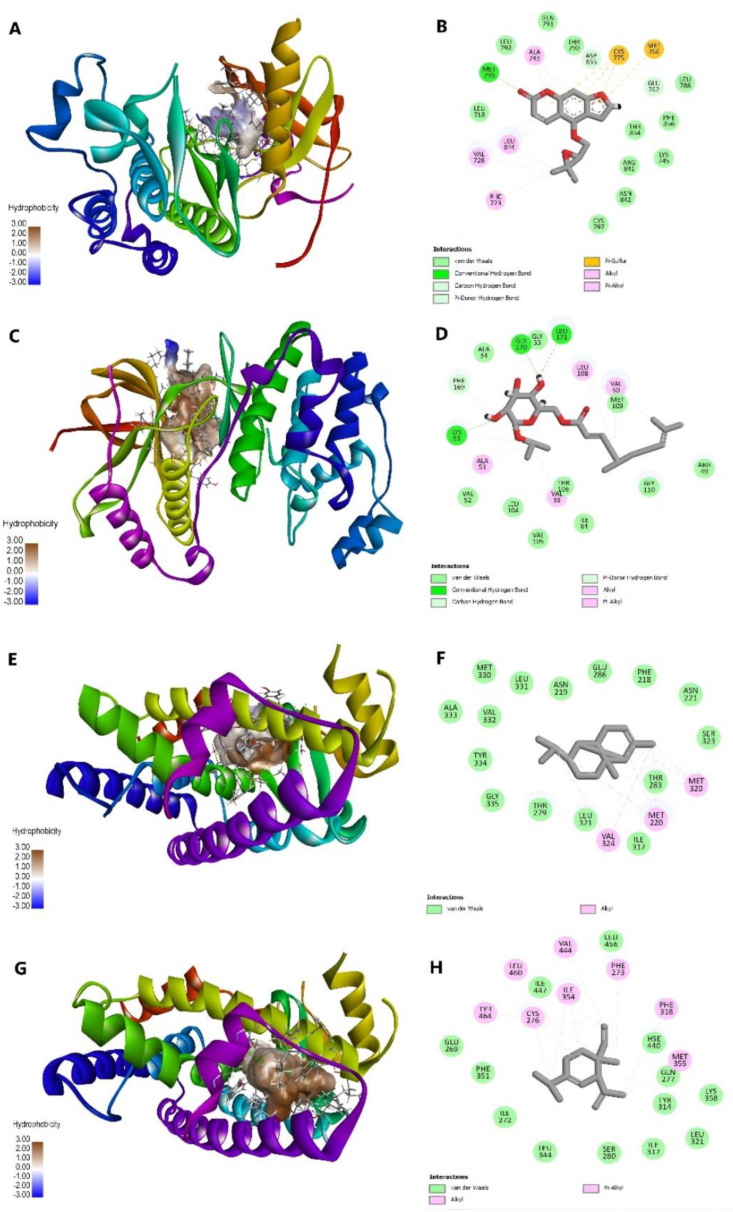
Attracting Cavities 2.0 docking visualization of EGFR–oxypeucedanin (A–B), MAPK–citrusoside A (C–D), PPARA–copaene (E–F), and PPARA–δ-elemene (G–H) using Discovery Studio. The visualization on the left shows the protein structure and its hydrophobicity, and the right picture reveals the residue interaction of the complex.

The binding interaction for complexes yielded from AC docking is illustrated in Fig. 5. Several of the interacting residues in AC-docked control complexes differed from those found in Vina-docked control complexes. However, some common residues were also identified, including CYS797 (hydrogen bond), LEU844, LEU718, and VAL726 (hydrophobic interaction) residues present in EGFR complex; PHE169, ALA51, ALA34, LYS 53, LEU108, and VAL38 (hydrophobic interaction) residues in MAPK14 complex; and almost all hydrogen bond and hydrophobic interaction residues in PPARA complex.

Based on the AC docking simulation, the EGFR–oxypeucedanin complex exhibited relatively low hydrophobicity and retained only one of the interacting residues in R28 that formed a hydrogen bond, specifically GLU762. However, it formed hydrogen bonds with different residues compared to the control, including MET93 and ASP855, indicating its potential affinity with the target. The MAPK14–citrusoside A complex showed greater hydrophobicity and maintained LYS53 as the interacting residue for hydrogen bond formation. Additionally, the ligand formed hydrogen bonds with GLY170, LEU171, and PHE169. Conversely, while the PPARA complexes exhibited overall good hydrophobicity, copaene and δ-elemene (the complexes with the highest binding affinity) did not form any hydrogen bonds when interacting with the target. Furthermore, the complex between PPARA and copaene showed no interacting residues similar to those in the control. In general, it was anticipated that the binding of PPARA with its associated compounds would be unstable (Fig. 5). The comprehensive residue interaction information for AC-docked complexes is available in Table S8.

### 3.6 Molecular dynamics simulation

EGFR—oxypeucedanin and MAPK14—citrusoside A were subsequently employed in a molecular dynamics investigation for 10 ns to confirm the stability of their bindings. The analysis encompassed three distinct parameters: RMSD Cα, RMSD ligand conformation, and RMSD ligand movement. RMSD plots of the EGFR—oxypeucedanin protein backbone Cα atoms exhibited comparable fluctuations with both EGFR—R28 and single EGFR, with RMSD ranging from 1.2 to 2.8 Å and a mean value of 2.4 Å. This average value is slightly lower than the control complex and single protein. A similar case was observed in the MAPK14—citrusoside A complex where the RMSD Cα value was even lower than that of the MAPK14—GE5 and single MAPK14 by 0.7–1.1 Å. This indicates that the protein structures bound to the chosen KL compounds might assume a more stable conformation compared to the control complexes (Figs. S5 and S6).

The RMSD measurements for the ligand's conformation and its movement away from the binding site over a 10 ns period were also examined. The RMSD ligand conformation of EGFR—oxypeucedanin exhibited significant fluctuations, but consistently stayed below 2.7 Å with an average value of 1.33 Å, slightly less than the control. This complex also showed substantial fluctuations in ligand movement at 4 ns, peaking at an RMSD of 5.209 Å. Nonetheless, it remained stable throughout the simulation with an average RSMD of 3.2 Å. This value is slightly greater than the control complex, with a mean RMSD of 2.45 Å. MAPK14—citrusoside A showed a slightly lower deviation in ligand conformation with an average value of 1.33 Å and consistent dynamics compared to control. Subsequently, this complex had a higher mean value of RMSD ligand movement compared to the control (4.37 < 5 Å). However, the movement demonstrated more stability with reduced fluctuations (Figs. S5 and S6).

## 4 Discussion

AD is a neurodegenerative, multifactorial disease with various facets. In managing such disease, natural products with their multitarget mechanisms are considered efficient drug candidates. One of the natural products exhibiting anti-AD properties is KL. Owing to its several bioactive compounds proven to have anti-Alzheimer activities (mediating Aβ-toxicity, inhibiting related targets e.g. acetylcholinesterase), such as sitosterol [21,[27], [28], [29]]. Recent research has also shown that KL peel extract exhibits a neuroprotective effect against oxidative stress [64] and improves spatial memory [65] in mice in vivo studies. However, it remained unclear how the compounds interact with AD targets and the possible aftereffects. For a comprehensive understanding of the complexity of KL multitarget mechanism, network pharmacology combined with molecular docking and molecular dynamics simulations is a useful bioinformatics tool to be used prior to a costly study. Here, the possible mechanism of KL in modulating AD symptoms was elucidated using network pharmacology, molecular docking, and molecular dynamics simulation.

In this study, databases and literature were utilized to determine the multitarget ability of KL compounds in affecting various factors of AD and the pathways involved in the process. A combined total of 69 substances present in the leaf, peel, and essential oil (extracted from leaf, branch, fruit peel, and blossom) of KL were initially detected across all sources before being reduced to 52 secondary metabolites. Secondary metabolites play a huge role in plants’ defense mechanism and have various pharmacological activities, making them important in drug discovery [[66], [67], [68], [69], [70]]. To assess the potential influence of these bioactive compounds on a range of proteins, they underwent a target identification process using SEA Search Server and STP. The SEA Server generates a wide range of potential targets using the chemical similarity ensemble approach and offers the P-value for each target produced, which corresponds to the P-value of the Z-score (raw Z-score under the reference SEA background null model) [35]. STP, on the other hand, utilizes 2D and 3D similarity measures to forecast the targets and employs probability scores for their ranking [36]. We selected only the top three ranked targets (or ten when all generated targets had equal scores) based on P-value scores for SEA Search Server and probability scores for STP from the range of targets produced by these algorithms. This approach ensured the inclusion of the most statistically significant targets, resulting in 127 primary targets predicted to be affected by KL secondary metabolites.

To investigate whether any of these targets were directly linked to AD, three databases (DisGeNET, OMIM, and GenCLiP3) were employed. After removing duplicates, a collective of 4647 genes associated with AD was identified. The comparison of both sets of genes revealed 64 AD-associated genes targeted by KL secondary metabolites, leading to the construction of a PPI network for mapping their interactions. Further investigation was required to comprehend how this network might play a role in biological pathways for creating an effective multitargeted drug. Therefore, KEGG pathway enrichment analysis was conducted to determine the mode of action of these genes in the network, and 14 statistically significant signaling pathways were found after being filtered by FDR. These encompass highly enriched pathways with notable rich factors and gene counts, indicating potential pathways involved in how KL could impact AD. Among these pathways, the inflammatory mediator regulation of TRP channels was the most enriched. TRP channels, which are protein membranes activated by various stimuli, have a strong correlation with inflammatory and oxidative activities, which lead to AD [[71], [72], [73], [74], [75], [76], [77], [78]]. These channels are involved in neuroinflammation and oxidative stress pathways induced by Aβ plaque accumulation, leading to neuronal death, which is the main feature of AD [71,73,75,76]. Therefore, inflammation and oxidative stress in AD could be attenuated by modulating a variety of implicated proteins. This finding suggested that KL may affect and play a role in AD pathophysiology in similar ways.

Further analysis of the 64 genes PPI network unveiled the top 10 core genes using the MCC algorithm. These genes could significantly impact the advancement of AD through modulation by KL secondary metabolites. For further elucidation, the top 10 genes were utilized for constructing a CTI network, revealing the primary ligand-target interactions. More than half of the total secondary metabolites targeted the most essential genes. Additionally, in a complete CTI network involving 64 genes and 52 secondary metabolites, nearly all KL secondary compounds targeted AD-associated genes. These findings indicate that KL could plausibly impact AD by influencing these targets through its secondary metabolites in a multitarget approach.

The network analysis results indicated the potential of KL secondary metabolites in influencing key genes. To confirm these findings, three targets were chosen from the analysis: EGFR, MAPK14, and PPARA. EGFR emerged as the highest-scoring target with significant potential. MAPK14 also exhibited a high MCC score, placing it among the top ten core genes in the PPI network. Moreover, this target was directly involved in the highest enriched pathway. PPARA was chosen due to its ranking among the top ten genes and being a target of multiple KL compounds. These three proteins are well-documented regarding their roles in inflammatory and oxidative stress pathways in AD, which further justifies their selection for deeper investigation. Although STAT3 and PRKCA showed promise as potential targets, they were excluded from the docking study due to issues related to validation and finding suitable structures respectively.

MAPK14/p38α is one of the mediators directly involved in regulating the inflammatory response of TRP channels and is also included in the top 10 most pivotal genes in the PPI network of this study. MAPK14 is a part of p38 kinases that mediate extracellular responses, particularly proinflammatory cytokines [79,80] and reactive oxygen species related to oxidative stress [[81], [82], [83], [84]]. It is highly activated in AD [[85], [86]]. A vast amount of research is focused on how MAPK14 is implicated in AD via multiform pathways; one of them being the modulator of the autophagy–lysosomal system dysfunction that leads to the aggregation and consequent impairment of the clearance of misfolded proteins (Aβ accumulation) [82,87,88]. Moreover, an in vivo study by Schnoder et al. (2016) showed that a low level of MAPK14 facilitates the lysosomal degradation of BACE1, resulting in the attenuation of amyloid pathology in AD. The mechanism is believed to be correlated with microglia cells, which are responsible for neuroinflammation in the brain, which in turn induces neurotoxicity and cognitive function loss [89]. Another in vivo study in a mouse model by Colie et al. (2017) [89] discovered that neuronal deletion of MAPK14 improves memory and synaptic plasticity. The high involvement of MAPK14 with AD factorial processes serves as a fundamental reason for its potential as an AD drug target.

EGFR and PPARA also exhibited high MCC scores and are involved in neuroinflammation and oxidative stress in AD. EGFR inhibition has been proposed as a therapeutic target for AD through several neuroprotective mechanisms, such as autophagy induction and reactive astrocyte attenuation leading to inflammation and Aβ accumulation [90,91]. In a similar context, PPARA has also been studied extensively and found to regulate genes related to many AD-associated biological processes, particularly inflammation and antioxidative defense [[92], [93], [94], [95], [96]]. In a recent in vivo study, the activation of PPARA led to anti-inflammatory and antioxidative activities and thereby significantly inhibited the progression of the disease [92]. Similar studies by Chandra and Pahan (2018) [97], Chandra et al. (2019) [98], and Luo et al. (2020) [99] found that PPARA activation decreased amyloid plaque pathology and improved memory, supporting the potential of PPARA as an AD therapeutic target.

All the above studies promoted the importance of the most essential proteins found in the current work for AD pathophysiology, particularly EGFR, MAPK14, and PPARA. To validate the network analysis result and further clarify the anti-AD potential of KL compounds, these proteins were docked with their corresponding compounds according to the previous target identification analysis. In addition, appropriate controls were utilized for comparative analysis. The selection included R28 and GE5 as small molecules known to bind to the active sites and inhibit the functions of EGFR and MAPK14 respectively, along with T06 identified as a PPARA activator. Prior to conducting the actual simulation with the metabolites, redocking was carried out between the protein and its native ligand (control) at the binding site, achieving an RMSD ≤2.0 Å for all redocked complexes. RMSD in the redocking study is used to measure the similarity between the molecular conformations of the predicted pose of docked ligands and the reference pose obtained from experimental crystallographic data. The higher the value, the higher the deviation of the predicted pose from the reference pose, making lower values more desirable [100,101]. Thus, this process confirmed the reliability and validity of the docking simulation.

Molecular docking was performed with two different docking engines, AutoDock Vina (PyRx) and AC 2.0 (SwissDock). The analysis included the binding energy and interactions formed by each complex. Binding affinity values represent the free energy of ligand binding, with lower values suggesting stronger and more stable interaction between the protein and ligand [63,102,103]. Complexes with a binding energy below −7 kcal/mol are considered more energetically favorable and thus predicted to have a strong binding [63]. Subsequently, several forces are implicated in the process of protein-ligand binding, including hydrogen bonding and hydrophobic interactions. Hydrogen bonds are typically the primary driver of affinity between the molecules; however, it is important to also take into account the impact of hydrophobicity or hydrophobic interactions on binding stability alongside hydrogen bonds [104,105]. EGFR—oxypeucedanin showed a strong affinity value in both docking simulations, although slightly lower than that of the control. Moreover, the complex retained a sufficient number of interacting residues in the control, forming hydrogen bonds and hydrophobic interactions. This suggests that EGFR—oxypeucedanin may have formed a stable binding at the active site of the target, potentially inhibiting EGFR in a manner similar to the inhibitor control.

The MAPK14—citrusoside A complex also displayed a strong predicted binding with a relatively large number of interacting residues involved in hydrogen bonds and hydrophobic interactions in both docking experiments. More hydrogen bonds were observed in the AC-docked results compared to Vina. Furthermore, the complex exhibited higher hydrophobicity compared to EGFR—oxypeucedanin, providing additional evidence of citrusoside A's potential to block MAPK14 through its binding site. Finally, the PPARA results indicated a slight variance between the two docking algorithms but remained generally consistent. According to Vina, β-caryophyllene and germacrene D exhibited the best binding energies, while copaene and δ-elemene were identified as top binders in AC. Despite exhibiting strong hydrophobic properties, the analysis indicated that these compounds did not engage in hydrogen bonding. As a result, it is anticipated that the bond between the protein and ligand will be fragile and unstable, potentially rendering the corresponding secondary metabolites ineffective in modulating PPARA activity.

Molecular dynamics simulations were conducted for 10 ns to confirm the binding stability of two best-docked complexes: EGFR—oxypeucedanin and MAPK14—citrusoside A. The evaluation included assessing the RMSD of protein backbone Cα atoms, ligand conformation, and movement from the binding pocket. RMSD is used in molecular dynamics studies to measure the distance between atoms over time and evaluate the structural stability of protein-ligand complexes [[106], [107], [108], [109]]. Good structural stability is indicated by low deviation or an RMSD of 1–4 Å [55]. EGFR—oxypeucedanin exhibited a more stable protein conformation compared to the control. The ligand also showed slightly greater stability than the control, with relatively low movement on average, indicating that it remains bonded in the protein's binding pocket and maintains a stable conformation. These findings confirm oxypeucedanin's potency in forming a stable binding with EGFR's active site and suppressing its functions.

On the other hand, MAPK14—citrusoside A exhibited a notably stable protein conformation, even compared to the single protein without any ligand intervention. Moreover, the ligand conformation displayed a higher stability than the control. The movement and conformation of the ligand were generally more stable with fewer fluctuations compared to the control. However, the average movement of the ligand reached 5 Å, indicating that it may potentially move away from the binding pocket despite stable structural conformations. Overall, the results suggest that citrusoside A has inhibitory potential towards MAPK14.

This study indicates that oxypeucedanin and citrusoside A show promise as inhibitors of EGFR and MAPK14, respectively. Inhibiting these genes could potentially reduce inflammation and oxidative stress, as they play a significant role in these interconnected biological processes. Inflammation and oxidative stress are seen as pivotal contributors to the onset of AD, representing a major risk factor for the disease [110]. Hence, it is logical to infer that blocking the core genes responsible for activating these processes could lead to successful prevention and/or treatment of AD. Yuan et al. (2023) [111] conducted a pertinent study that showcased the effectiveness of a novel multitarget drug, Zn^2+^-responsive palladium nanoclusters, in addressing neuroinflammation and oxidative stress in AD. The drug demonstrated success in neutralizing reactive oxygen species to reduce oxidative stress and regulating microglial polarization to decrease neuroinflammation, ultimately leading to a neuroprotective outcome and enhancing cognitive function in an AD mice model [111]. This suggests that oxypeucedanin and citrusoside A could potentially yield a comparable result due to their predicted inhibitory impact on these physiological activities.

However, till date, investigations on oxypeucedanin and citrusoside A are scarce. One correlating study reported the anti-inflammatory activity of oxypeucedanin in LPS-induced acute lung injury in vitro and in vivo models [112]. Another study by Karakaya et al. (2019) [113] found that oxypeucedanin isolated from parts of *Angelica purpurascens* also had high antioxidant and anticholinesterase activities, thereby supporting the results of our study. Acetylcholinesterase (AChE) has been proposed as a potential target for AD treatment due to evidence showing that inhibiting its activities can lead to improvement in the condition [[114], [115], [116]]. However, our investigation did not indicate any interaction between oxypeucedanin and AChE, so we did not explore this further.

As shown in the CTI network, oxypeucedanin and citrusoside A targeted many other proteins related to AD. Hence, they may affect AD via pathways other than inflammation and oxidative stress. Furthermore, these compounds may work synergistically with all other KL secondary metabolites (except α-bergamotene) to regulate the 64 AD targets in a multitarget, multipathway mechanism. However, it is important to acknowledge that controlling the activity of these proteins could lead to alterations in additional pathways not addressed in this research. For instance, EGFR and MAPK14 are protein kinases present throughout the body, playing diverse roles in various conditions. EGFR has been recognized as a notable therapeutic target for several types of cancers, particularly carcinomas [[117], [118], [119], [120]]. On the other hand, the involvement of the MAPK pathway in cell proliferation and inflammation adds significant complexity, particularly concerning drug development strategies [121]. This illustrates the potential use of these KL substances for alternative applications, such as creating medications for cancer, antioxidants, or anti-inflammatory purposes. However, it also presents a challenge in terms of precision, posing a common issue in the development of various types of drugs. Therefore, more comprehensive research is necessary to verify the accuracy and effectiveness of the multi-targeted anti-AD drug.

Lastly, there are other constraints to this investigation. It relies on multiple databases, and the outcomes could vary if those same databases were accessed at different times. Furthermore, our research is entirely computational, and despite attempts to replicate a natural environment, real-life results may differ. These represent the challenges encountered in network pharmacology. Nevertheless, this approach continues to show potential in overcoming the limitations of the "one drug, one target" paradigm. Consequently, it is crucial to validate the findings of this study through in vitro and in vivo methods in future investigations.

## 5 Conclusion

This computational study revealed that oxypeucedanin and citrusoside A derived from KL showed promise in inhibiting EGFR and MAPK14 activities respectively. These targets are closely associated with inflammatory and oxidative stress pathways, revealing the possible mechanism of KL in attenuating AD symptoms. Further work is needed to validate the results using other methods, such as in vitro or in vivo studies.

## Funding

Not applicable.

## Data availability statement

Raw data, model structure, and trajectory analysis are available upon request.

## CRediT authorship contribution statement

**Adhisa Fathirisari Putri:** Writing – original draft, Software, Formal analysis, Data curation, Conceptualization, Investigation, Methodology, Project administration, Validation, Visualization, Writing – review & editing. **Didik Huswo Utomo:** Writing – review & editing, Supervision, Resources, Methodology, Conceptualization. **Woro Anindito Sri Tunjung:** Supervision, Resources. **Wahyu Aristyaning Putri:** Writing – review & editing, Supervision, Resources, Funding acquisition.

## Declaration of competing interest

The authors, Adhisa Fathirisari Putri, Didik Huswo Utomo, Woro Anindito Sri Tunjung, and Wahyu Aristyaning Putri, declare no conflicts of interest associated with the research study titled "Analysis of the Anti-Alzheimer Potential of Bioactive Compounds from Citrus hystrix DC. Peel, Leaf, and Essential Oil by Network Pharmacology," submitted to Heliyon.
