# Supplementary material for: Analysis of the anti-Alzheimer potential of bioactive compounds from Citrus hystrix DC. peel, leaf, and essential oil by network pharmacology
Source: Heliyon. 2024 Jun 26;10(13):e33496. doi: 10.1016/j.heliyon.2024.e33496 (PMC11267028; doi:10.1016/j.heliyon.2024.e33496)
Supplement: Multimedia component 1 [file mmc1.pdf]

Table S1. Total compounds identified from *Citrus hystrix* DC. The non-highlighted compounds were determined as secondary metabolites and chosen for further analysis.

No.	Compound	Source	Reference
1	*trans*-β-Farnesene	Blossom (flower) extract (volatile)	KNApSAcK - Jabalpurwala (2009)
2	α-Thujene	Blossom (flower) extract (volatile)	KNApSAcK - Jabalpurwala (2009)
3	Hexanal (fatty aldehyde)	Blossom (flower) extract (volatile)	KNApSAcK - Jabalpurwala (2009)
4	Hexanol	Blossom (flower) extract (volatile)	KNApSAcK - Jabalpurwala (2009)
5	α-Pinene	Blossom (flower) extract (volatile)<br>Fruit peel essential oil	KNApSAcK - Jabalpurwala (2009)<br>Warsito (2017)
6	β-Pinene	Blossom (flower) extract (volatile)<br>Leave, fruit peel, and bark essential oil	KNApSAcK - Jabalpurwala (2009)<br>Warsito (2017)
7	Limonene	Blossom (flower) extract (volatile)<br>Bark, leave, and fruit peel essential oil	KNApSAcK - Jabalpurwala (2009)<br>Warsito (2017)
8	β-Myrcene	Blossom (flower) extract (volatile)<br>Bark, leave, and fruit peel essential oil	KNApSAcK - Jabalpurwala (2009)<br>Warsito (2017)
9	α-Terpinolene	Blossom (flower) extract (volatile)	KNApSAcK - Jabalpurwala (2009)
10	Pentadecane	Blossom (flower) extract (volatile)	KNApSAcK - Jabalpurwala (2009)
11	Indole	Blossom (flower) extract (volatile)	KNApSAcK - Jabalpurwala (2009)
12	2-Phenylethyl alcohol	Blossom (flower) extract (volatile)	KNApSAcK - Jabalpurwala (2009)
13	4-Cymene	Blossom (flower) extract (volatile)<br>Bark essential oil	KNApSAcK - Jabalpurwala (2009)<br>Warsito (2017)
14	Linalool	Blossom (flower) extract (volatile)	KNApSAcK - Jabalpurwala (2009)
15	α-Terpinene	Blossom (flower) extract (volatile)<br>Fruit peel essential oil	KNApSAcK - Jabalpurwala (2009)<br>Warsito (2017)
16	β-Caryophyllene	Blossom (flower) extract (volatile)<br>Leave, fruit peel extract<br>Bark, leave, and fruit peel essential oil	KNApSAcK - Jabalpurwala (2009)<br>Pattarachotanant & Tencomnao (2020)<br>Warsito (2017)
17	Copaene	Blossom (flower) extract (volatile)<br>Fruit peel extract<br>Fruit peel essential oil	KNApSAcK - Jabalpurwala (2009)<br>Pattarachotanant & Tencomnao (2020)

			Warsito (2017)
18	β-Bisabolene	Blossom (flower) extract (volatile)	KNApSAcK - Jabalpurwala (2009)
19	β-Elemene	Blossom (flower) extract (volatile)	KNApSAcK - Jabalpurwala (2009)
20	δ-3-Carene	Blossom (flower) extract (volatile)	KNApSAcK - Jabalpurwala (2009)
21	δ-Elemene	Blossom (flower) extract (volatile)	KNApSAcK - Jabalpurwala (2009)
22	Ethanol	Blossom (flower) extract (volatile)	KNApSAcK - Jabalpurwala (2009)
23	Calamenene	Blossom (flower) extract (volatile)	KNApSAcK - Jabalpurwala (2009)
24	Sabinene	Blossom (flower) extract (volatile)<br>Bark, leave, and fruit peel essential oil	KNApSAcK - Jabalpurwala (2009)<br>Warsito (2017)
25	Methyl anthranilate	Blossom (flower) extract (volatile)	KNApSAcK - Jabalpurwala (2009)
26	6-Methyl-5-hepten-2-one	Blossom (flower) extract (volatile)	KNApSAcK - Jabalpurwala (2009)
27	(*E*)-Ocimene	Blossom (flower) extract (volatile)<br>Bark, leave essential oil	KNApSAcK - Jabalpurwala (2009)<br>Warsito (2017)
28	Citronellal	Blossom (flower) extract (volatile)<br>Leave, fruit peel extract<br>Bark, leave, and fruit peel essential oil	KNApSAcK - Jabalpurwala (2009)<br>Pattarachotanant & Tencomnao (2020)<br>Warsito (2017)
29	2,6-Dimethyl-1,3,5,7-octatetraene (Cosmene)	Blossom (flower) extract (volatile)	KNApSAcK - Jabalpurwala (2009)
30	2-Methylfuran	Blossom (flower) extract (volatile)	KNApSAcK - Jabalpurwala (2009)
31	Acetone	Blossom (flower) extract (volatile)	KNApSAcK - Jabalpurwala (2009)
32	2,6-Dimethyl-2,4,6-octatriene (Neoalloocimene)	Blossom (flower) extract (volatile)	KNApSAcK - Jabalpurwala (2009)
33	α-Bergamotene	Blossom (flower) extract (volatile)	KNApSAcK - Jabalpurwala (2009)
34	α-Myrcene	Blossom (flower) extract (volatile)	KNApSAcK - Jabalpurwala (2009)
35	Benzene acetonitrile	Blossom (flower) extract (volatile)	KNApSAcK - Jabalpurwala (2009)
36	Carvyl acetate	Blossom (flower) extract (volatile)	KNApSAcK - Jabalpurwala (2009)
37	Isopropyl alcohol	Blossom (flower) extract (volatile)	KNApSAcK - Jabalpurwala (2009)
38	Methyl acetate	Blossom (flower) extract (volatile)	KNApSAcK - Jabalpurwala (2009)

39	Sabinene hydrate	Blossom (flower) extract (volatile)	KNApSAcK - Jabalpurwala (2009)
40	Citrusoside A	Fruit peel extract	KNApSAcK – Youkwan (2010)
41	Citrusoside B	Fruit peel extract	KNApSAcK – Youkwan (2010)
42	Citrusoside C	Fruit peel extract	KNApSAcK – Youkwan (2010)
43	Citrusoside D	Fruit peel extract	KNApSAcK – Youkwan (2010)
44	Sitosterol	Leave, fruit peel extract	Pattarachotanant & Tencomnao (2020)
45	Citronellol	Leave, fruit peel extract<br>Bark essential oil	Pattarachotanant & Tencomnao (2020)<br>Warsito (2017)
46	α-Terpineol	Fruit peel extract<br>Bark, fruit peel extract	Pattarachotanant & Tencomnao (2020)
47	Methyl-6-oxoheptanoate	Fruit peel extract	Pattarachotanant & Tencomnao (2020)
48	β-Cubebene	Fruit peel extract	Pattarachotanant & Tencomnao (2020)
49	Cadinene	Fruit peel extract<br>Leave, fruit peel essential oil	Pattarachotanant & Tencomnao (2020)<br>Warsito (2017)
50	Phytol	Leave, fruit peel extract	Pattarachotanant & Tencomnao (2020)
51	Phenol, 2,4-bis(1,1-dimethylethyl)-	Leave, fruit peel extract	Pattarachotanant & Tencomnao (2020)
52	7-Hydroxycoumarin (Umbelliferone)	Fruit peel extract	Pattarachotanant & Tencomnao (2020)
53	n-Hexadecanoic acid	Leave, fruit peel extract	Pattarachotanant & Tencomnao (2020)
54	*7H*-Furo(3,2-g)(1)benzopyran-7-one, 9-hydroxy- (Xanthotoxol)	Fruit peel extract	Pattarachotanant & Tencomnao (2020)
55	*7H*-Furo(3,2-g)(1)benzopyran-7-one, 4-(2,3-epoxy-3-methylbutoxy)-, (*S*)-(-)- (Oxypeucedanin)	Leave, fruit peel extract	Pattarachotanant & Tencomnao (2020)
56	Cyclohexanol, 2-(2-hydroxy-2-propyl)-5-methyl-	Leave extract	Pattarachotanant & Tencomnao (2020)
57	α-Phellandrene	Fruit peel extract	Warsito (2017)
58	4-(3-Methyl-2-oxobutoxy)-*7H*-furo(3,2-g)(1)benzopyran-7-one (Isooxypeucedanin)	Fruit peel extract	Pattarachotanant & Tencomnao (2020)
59	γ-Terpinene	Bark, fruit peel essential oil	Warsito (2017)
60	Epoxy-linalool oxide	Bark, leave, and fruit peel essential oil	Warsito (2017)
61	Isopulegol	Bark essential oil	Warsito (2017)
62	Rhodinol	Bark, fruit peel essential oil	Warsito (2017)
63	Linalyl oxide	Bark essential oil	Warsito (2017)

64	Citronellyl acetate	Bark, leave essential oil	Warsito (2017)
65	Geranyl acetate	Bark, leave, and fruit peel essential oil	Warsito (2017)
66	Cyclo-Germacrene (Germacrene-D)	Leave essential oil	Warsito (2017)
67	Nerolidol	Leave extract<br>Bark essential oil	Pattarachotanant & Tencomnao (2020)<br>Warsito (2017)
68	4-(2,3-Dihydroxy-3-methylbutoxy)furo(3,2-g)chromen-7-one (Oxypeucedanin hydrate)	Leave, fruit peel extract	Pattarachotanant & Tencomnao (2020)
69	1,2-Benzenedicarboxylic acid, butyl octyl ester	Leave extract	Pattarachotanant & Tencomnao (2020)

Table S2. Target identification of 52 secondary metabolites of *Citrus hystrix* DC. peel, leaf, and essential oil by Swiss Target Prediction and SEA Server.

No	Compound	Swiss Target Prediction	Probability	SEA Server	P-value	MaxTC
1	trans-β-Farnesene	no target predicted	-	Lanosterol synthase<br>Squalene epoxidase (NONHUMAN)<br>Geranylgeranyl pyrophosphate synthase	4.172e-19<br>1.611e-73<br>4.339e-58	0.46<br>0.52<br>0.52
2	α-Thujene	Peroxisome proliferator-activated receptor alpha<br>Cannabinoid receptor 2<br>Nuclear receptor subfamily 1 group I member 3 (by homology)	0.0743164735672<br>0.0743164735672<br>0.0238327432783	Heat sensitive channel TRPV3 (RAT)	1.665e-08	0.28
3	α-Pinene	Peroxisome proliferator-activated receptor alpha<br>Cannabinoid receptor 2<br>Acetylcholinesterase	0.0649510872641<br>0.0649510872641<br>0.0544879468076	C-X-C chemokine receptor type 3<br>Tyrosyl-DNA phosphodiesterase 1	2.54e-27<br>8.882e-16	0.40<br>0.36
4	β-Pinene	High-affinity choline transporter (by homology)<br>11-beta-hydroxysteroid dehydrogenase 1<br>UDP-glucuronosyltransferase 2B7	0	no results		
5	Limonene	Peroxisome proliferator-activated receptor alpha<br>Cannabinoid receptor 2<br>LXR-alpha	0.146856850103<br>0.146856850103<br>0.0443083186041	no results		
6	β-Myrcene	Peroxisome proliferator-activated receptor alpha	0.0544879468076<br>0.0544879468076	Squalene epoxidase (PIG)<br>Squalene monooxygenase	2.35e-55<br>7.838e-37	0.39<br>0.39<br>0.29

		Cannabinoid receptor 2<br>Testis-specific androgen-binding protein	0	Squalene synthase (YEAST)	3.875e-36	
7	α-Terpinolene	Peroxisome proliferator-activated receptor alpha<br>Cannabinoid receptor 2<br>Zinc finger protein GLI2	0.0443083186041<br>0.0443083186041<br>0	no results		
8	Pentadecane	no target prediction		CAI-1 autoinducer sensor kinase/phosphatase CqsS<br>Phospholipase B<br>Carboxylic ester hydrolase<br>(ALL NONHUMAN)	5.509e-117<br>5.964e-114<br>2.258e-87	0.44<br>0.62<br>0.38
9	4-Cymene	Cytochrome P450 2A6<br>Acetylcholinesterase<br>Trace amine-associated receptor 1	0.0443083186041<br>0.0238327432783<br>0.0238327432783	Reverse transcriptase (EUKARYOTE)<br>Phospholipase A2 (RAT)<br>NACHT, LRR and PYD domains-containing protein 1	5.965e-45<br>6.772e-15<br>4.43e-14	0.36<br>0.28<br>0.31
10	Linalool	Transient receptor potential cation channel subfamily V member 3<br>Carbonic anhydrase II<br>Carbonic anhydrase I	0.0630257148888	Squalene monooxygenase<br>Squalene epoxidase (PIG)<br>Geranylgeranyl pyrophosphate synthase	5.719e-31<br>3.081e-26<br>7.666e-20	0.32<br>0.32<br>0.32
11	α-Terpinene	Peroxisome proliferator-activated receptor alpha<br>Cannabinoid receptor 2<br>Adenosine A1 receptor	0	no results		
12	β-Caryophyllene	Peroxisome proliferator-activated receptor alpha<br>Cannabinoid receptor 2	0.719173986015<br>0.719173986015<br>0.0428942118511	Heat sensitive channel TRPV3 (RAT)<br>Zinc finger protein GLI2<br>Zinc finger protein GLI1	1.44e-44<br>5.551e-16<br>1.321e-08	0.35<br>0.30<br>0.30

		Anandamide amidohydrolase				
13	Copaene	Peroxisome proliferator-activated receptor alpha<br>Cannabinoid receptor 2<br>Androgen Receptor (by homology)	0.109507900685<br>0.109507900685<br>0.0428942118511	no results		
14	β-Bisabolene	no target predicted		Squalene epoxidase (PIG)<br>Lanosterol synthase (RAT)<br>Phospholipase A2 (EUKARYOTE)	2.158e-24<br>4.947e-21<br>1.485e-17	0.30<br>0.35<br>0.29
15	β-Elemene	C-X-C chemokine receptor type 3<br>Serotonin 2a (5-HT2a) receptor<br>Monoamine oxidase B	0.0428942118511<br>0<br>0	no results		
16	δ-3-Carene	Peroxisome proliferator-activated receptor alpha<br>Cannabinoid receptor 2<br>Anandamide amidohydrolase	0.0649510872641<br>0.0649510872641<br>0.0238327432783	Cannabinoid receptor 2 (MOUSE)<br>Cannabinoid receptor 1 (RAT)	1.44e-19<br>1.551e-10	0.33<br>0.33
17	δ-Elemene	Peroxisome proliferator-activated receptor alpha<br>Cannabinoid receptor 2<br>LXR-alpha	0.0524239602665<br>0.0524239602665<br>0.0428942118511	no results		
18	Calamenene	Androgen Receptor (by homology)<br>Cannabinoid receptor 1 (by homology)<br>Cannabinoid receptor 2	0.0524239602665<br>0.0428942118511<br>0.0428942118511	no results		
19	Sabinene	11-beta-hydroxysteroid dehydrogenase 1	0	no results		

		UDP-glucuronosyltransferase 2B7<br>Peroxisome proliferator-activated receptor alpha<br>Cannabinoid receptor 2<br>Acetylcholinesterase				
20	Methyl anthranilate	Carbonic anhydrase II<br>Carbonic anhydrase I<br>Huntingtin	0.0630257148888<br>0.0630257148888<br>0.053517944289	Tumor necrosis factor receptor superfamily member 6 (RAT)<br>Acetolactate synthase catalytic subunit (mitochondrial) (YEAST)<br>Kynureninase	2.316e-53<br>7.29e-50<br>5.333e-44	0.41<br>0.37<br>0.42
21	(*E*)-Ocimene	Peroxisome proliferator-activated receptor alpha<br>Cannabinoid receptor 2<br>Monoamine oxidase B<br>Acetylcholinesterase	0.0443083186041<br>0.0443083186041<br>0<br>0	no results		
22	Citronellal	Anandamide amidohydrolase<br>Cytochrome P450 19A1<br>Vanilloid receptor	0.101228839251<br>0.0918391658524<br>0.0727163610114	Macrophage scavenger receptor types I and II<br>Squalene synthase (RAT)<br>Geranylgeranyl pyrophosphate synthase	6.315e-44<br>2.447e-21<br>1.595e-12	0.35<br>0.46<br>0.35
23	2,6-Dimethyl-1,3,5,7-octatetraene (Cosmene)	Peroxisome proliferator-activated receptor alpha<br>Cannabinoid receptor 2<br>Testis-specific androgen-binding protein	0.0544879468076<br>0.0544879468076<br>0.0238327432783	no results		
24	2,6-Dimethyl-2,4,6-octatriene (Neoalloocimene)	Peroxisome proliferator-activated receptor alpha<br>Cannabinoid receptor 2	0.0443083186041<br>0.0443083186041<br>0	no results		

		Testis-specific androgen-binding protein				
25	α-Bergamotene	no target predicted		no results		
26	α-Myrcene	Peroxisome proliferator-activated receptor alpha<br>Cannabinoid receptor 2<br>Serotonin 2a (5-HT2a) receptor	0	no results		
27	Carvyl acetate	Cytochrome P450 19A1<br>HMG-CoA reductase (by homology)<br>Monoamine oxidase A	0.0619149311119<br>0.0414702990123<br>0.0414702990123	no results		
28	Sabinene hydrate	Transient receptor potential cation channel subfamily M member 8<br>Carbonic anhydrase II<br>Carbonic anhydrase I, IV	0.2934139092<br>11<br>0.274196023988<br>0.274196023988	no results		
29	Citrusoside A	**57 targets with the same probability**<br>Sodium/potassium-transporting ATPase alpha-1 chain<br>Signal transducer and activator of transcription 3<br>Protein kinase C alpha<br>Nuclear receptor ROR-gamma<br>MAP kinase p38 alpha<br>Adenosine kinase<br>Phospholipase A2 group 1B<br>Sodium/glucose cotransporter 1	0.115736675	Envelope glycoprotein gp160 (VIRUS)		

		Adenosine A2a receptor<br>Beta-glucosidase				
30	Citrusoside B	Adenosine A3 receptor<br>Matrix metalloproteinase 13<br>Sodium/glucose cotransporter 2	0.0498457217793<br>0.0498457217793<br>0	Acyl carrier protein (mitochondrial)<br>Complex I intermediate-associated protein 30 (mitochondrial)<br>NADH dehydrogenase [ubiquinone] 1 alpha subcomplex subunit 1	2.608e-66	0.45
31	Citrusoside C	**22 targets with the same probability**<br>Carbonic anhydrase I<br>Carbonic anhydrase XII<br>Carbonic anhydrase IX<br>Carbonic anhydrase II<br>Carbonic anhydrase VII<br>Neurokinin 2 receptor<br>Sodium/nucleoside cotransporter 2<br>Adenosine A2a receptor<br>Adenosine A3 receptor<br>Carbonic anhydrase XIII	0.035898205	Acyl carrier protein<br>Complex I intermediate-associated protein 30<br>NADH dehydrogenase [ubiquinone] 1 alpha subcomplex subunit 1 (mitochondrial)	4.092e-48	0.32
32	Citrusoside D	**37 targets with the same probability**<br>Carbonic anhydrase XIII<br>Carbonic anhydrase I<br>Carbonic anhydrase XII<br>Carbonic anhydrase IX<br>Carbonic anhydrase II<br>Neurokinin receptor	0.035898205	Acyl carrier protein<br>Complex I intermediate-associated protein 30<br>NADH dehydrogenase [ubiquinone] 1 alpha subcomplex subunit 1 (mitochondrial)	2.565e-45	0.30

		Adenosine A2a receptor<br>Adenosine A2b receptor<br>Adenosine A3 receptor<br>Eukaryotic initiation factor 4A-I				
33	Sitosterol	HMG-CoA reductase<br>Cytochrome P450 51 (by homology)<br>Androgen Receptor (EXCEL)	0.680955494<br>0.680955494<br>0.656003572	Prothrombin<br>Delta(24)-sterol reductase<br>Steroid 17-alpha-hydroxylase/17,20 lyase	0.9968<br>7.597e-103<br>4.865e-83	1.00<br>0.73<br>0.68
34	Citronellol	Androgen Receptor<br>Cytochrome P450 19A1<br>Squalene monooxygenase	0.0822880706974<br>0.0630257148888<br>0.0630257148888	Macrophage scavenger receptor types I and II<br>Lanosterol synthase (EUKARYOTES)<br>Squalene monooxygenase	5.166e-42<br>7.142e-37<br>1.858e-34	0.33<br>0.29<br>0.38
35	α-Terpineol	Androgen Receptor<br>Cytochrome P450 19A1<br>Carbonic anhydrase II	0.120677312392<br>0.0918391658524<br>0.0918391658524	no results		
36	β-Cubebene	C-X-C chemokine receptor type 3<br>11-beta-hydroxysteroid dehydrogenase 1<br>UDP-glucuronosyltransferase 2B7	0.0428942118511	no results		
37	Cadinene	Peroxisome proliferator-activated receptor alpha<br>Cannabinoid receptor 2<br>Androgen Receptor (by homology)	0.0714808399531<br>0.0714808399531<br>0.0428942118511	no results		
38	Phytol	**22 targets with the same probability**	0.0978745343258	Vitamin K epoxide reductase complex subunit 1 (EUKARYOTES)	4.331e-29<br>2.669e-26	0.47<br>0.32<br>0.38<br>0.32

		Dual specificity phosphatase Cdc25A<br>Androgen Receptor<br>UDP-glucuronosyltransferase 2B7<br>Dual specificity phosphatase Cdc25B<br>Glycine receptor subunit alpha-1<br>Protein kinase C gamma<br>Protein kinase C delta<br>Protein kinase C epsilon<br>Protein kinase C eta<br>Protein kinase C theta		Squalene epoxidase (PIG)<br>Lanosterol synthase (RAT)	5.063e-24<br>4.419e-21	
39	Phenol, 2,4-bis(1,1-dimethylethyl)-	Carbonic anhydrase II<br>Cyclooxygenase-1 (PTGS1)<br>Serotonin 2b (5-HT2b) receptor (HTR2B)<br>Seretonin 2c (5-HT2c) receptor (HTR2C)	0.204771116708<br>0.128639647928<br>0.119061923007<br>0.119061923007	Cytochrome b-c1 complex subunit 7 (QCR7)<br>cAMP-regulated phosphoprotein 19 (ARP19) (RAT)<br>Protein GrpE (GRPE) (E. coli)<br>Estrogen-related receptor gamma (ESRRG)<br>Acetylcholinesterase (AChE) (Eukaryotes)	1.333e-45<br>6.377e-23<br>1.523e-18<br>3.119e-13<br>4.75e-13	0.32<br>0.28<br>0.29<br>0.38<br>0.31
40	7-Hydroxycoumarin (Umbelliferone)	Carbonic anhydrase XII<br>Carbonic anhydrase IX<br>Epidermal growth factor receptor erbB1 (EXCEL)	0.989975593<br>0.989975593<br>0.265882416	Zn finger protein (EUKARYOTE)<br>Carbonic anhydrase 12<br>Carbonic anhydrase 9	3.629e-103<br>5.095e-22<br>2.084e-21	1.00
41	*7H*-Furo(3,2-g)(1)benzopyran-7-one, 9-hydroxy-(Xanthotoxol)	Acetylcholinesterase<br>Cytochrome P450 1A2<br>D-amino-acid oxidase	0.0714808399531<br>0.0714808399531<br>0.0524239602665	Zn finger protein (EUKARYOTES)<br>DNA polymerase iota<br>DNA polymerase eta<br>Carbonic anhydrase 13	2.233e-91<br>2.322e-19<br>3.331e-16	0.44<br>0.31<br>0.31<br>0.53

					8.771e-15	
42	*7H*-Furo(3,2-g)(1)benzopyran-7-one, 4-(2,3-epoxy-3-methylbutoxy)-, (*S*)-(-)-(Oxypeucedanin)	**101 targets with the same probability**<br>Melatonin receptor 1A<br>Melatonin receptor 1B<br>Cytochrome P450 19A1<br>Leucine-rich repeat serine/threonine-protein kinase 2<br>Gamma-secretase<br>Epidermal growth factor receptor erbB1<br>Androgen Receptor<br>Phosphodiesterase 4A<br>Phosphodiesterase 4B<br>Phosphodiesterase 4D	0.097874534	no results		
43	α-Phellandrene	Peroxisome proliferator-activated receptor alpha<br>Cannabinoid receptor 2<br>Adenosine A1, A2a, dan A3 receptor	0.0238327432783 1	no results		
44	4-(3-Methyl-2-oxobutoxy)-*7H*-furo(3,2-g)(1)benzopyran-7-one (Isooxypeucedanin)	Voltage-gated potassium channel subunit Kv1.5<br>Carbonic anhydrase IX<br>Androgen Receptor	0.155528102326<br>0.114337558605<br>0.106099949133	Potassium voltage-gated channel subfamily A member 3<br>Potassium voltage-gated channel subfamily A member 7<br>Potassium voltage-gated channel subfamily A member 1	4.165e-78<br>1.267e-68<br>3.179e-48	0.58<br>0.55<br>0.55
45	γ-Terpinene	Vanilloid receptor<br>Serine/threonine-protein kinase PLK1	0.0238327432783	no results		

		Zinc finger protein GLI2<br>Zinc finger protein GLI1				
46	Isopulegol	Carbonic anhydrase II<br>Carbonic anhydrase I<br>Carbonic anhydrase IV	0.101228839251	no results		
47	Rhodinol	11-beta-hydroxysteroid dehydrogenase 1<br>Cyclooxygenase-1<br>UDP-glucuronosyltransferase<br>2B7	0.0822880706974<br>0.0727163610114<br>0.0727163610114	no results		
48	Citronellyl acetate	Taste receptor type 2 member 31<br>Anandamide amidohydrolase<br>11-beta-hydroxysteroid dehydrogenase<br>1	0.0414702990123	Macrophage scavenger receptor types I and II<br>Squalene monooxygenase (RAT)<br>Transient receptor potential cation channel subfamily A member 1 (RAT)	2.905e-40<br>1.195e-11<br>2.246e-10	0.32<br>0.31<br>0.60
49	Geranyl acetate	**100 targets with the same probability**<br>Neuropeptide Y receptor type 5<br>Cytochrome P450 17A1<br>Epoxide hydrolase 1<br>11-beta-hydroxysteroid dehydrogenase 1<br>Acetylcholinesterase<br>Arachidonate 5-lipoxygenase<br>Histamine H3 receptor<br>Cytochrome P450 11B1<br>Cytochrome P450 11B2	0.0312265582077	Geranylgeranyl pyrophosphate synthase<br>Squalene epoxidase (PIG)<br>Alternative oxidase (mitochondrial) (EUKARYOTE)	3.553e-67<br>3.308e-65<br>1.908e-61	0.44<br>0.45<br>0.37

		Proteinase-activated receptor 1				
50	Cyclo-Germacrene (Germacrene D)	Peroxisome proliferator-activated receptor alpha<br>Cannabinoid receptor 2<br>Adenosine A1 receptor	0.0524239602665<br>0.0524239602665<br>0.0428942118511	Heat sensitive channel TRPV3 (RAT)	1.508e-26	0.31
51	Nerolidol	Squalene monooxygenase<br>Indoleamine 2,3-dioxygenase<br>Period circadian protein homolog 2	0.112450964818<br>0.0604245879294<br>0.0604245879294	Geranylgeranyl pyrophosphate synthase<br>Alternative oxidase, mitochondrial (EUKARYOTES)<br>Squalene monooxygenase<br>Lanosterol synthase	2.63e-59<br>1.54e-56<br>1.1e-44<br>5.551e-16	0.45<br>0.33<br>0.47<br>0.40
52	4-(2,3-Dihydroxy-3-methylbutoxy)furo(3,2-g)chromen-7-one (Oxypeucedanin hydrate)	no target predicted		Potassium voltage-gated channel subfamily A member 3<br>Potassium voltage-gated channel subfamily A member 7<br>Acyl carrier protein (mitochondrial)	1.707e-70<br>4.557e-66<br>1.191e-48	0.56<br>0.53<br>0.33

Table S3. MCC score of 64 target proteins in the protein-protein interaction network by Cytohubba.

**Protein**	**MCC**
EGFR	58
STAT3	54
TRPV1	39
PRKCA	32
CNR1	31
MAPK14	28
PRKCE	26
PPARA	26
AR	23
FAAH	21
TRPA1	14
PRKCG	14
PRKCD	13
GLI1	12
CNR2	12
CDC25A	10
MAOB	9
ADORA2A	8
FAS	8
ADORA1	8
MAOA	7
MTNR1B	7
CDC25B	7
ACHE	7
HTT	7
LRRK2	7
GLI2	7
MTNR1A	6
HTR2A	5
IDO1	5
CXCR3	5
HMGCR	4

**Protein**	**MCC**
PLA2G1B	4
CYP2A6	4
F2	3
PLK1	3
SQLE	3
PSEN2	3
NR1H3	2
PLB1	2
DHCR24	2
PDE4A	2
MMP13	2
PRKCH	2
CA2	2
KYNU	2
HSD11B1	2
PDE4B	2
KCNA3	2
PER2	2
CA1	2
VKORC1	2
PTGS1	2
EIF4A1	1
SHBG	1
SLC5A2	1
DAO	1
TDP1	1
SLC5A7	1
PDE4D	1
NR1I3	1
MSR1	0
TACR2	0
NLRP1	0

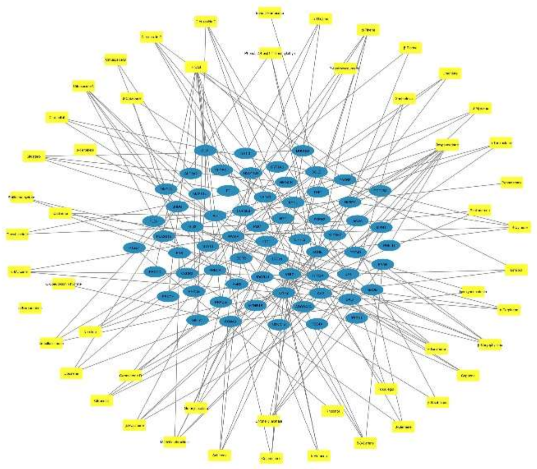

Figure S1. Complete CTI Network based on target identification results constructed with Cytoscape v.3.9.1. The genes are represented with blue circular nodes and the compounds are shown as yellow rectangular nodes. The edges reveal the interaction between each compound and its targets.

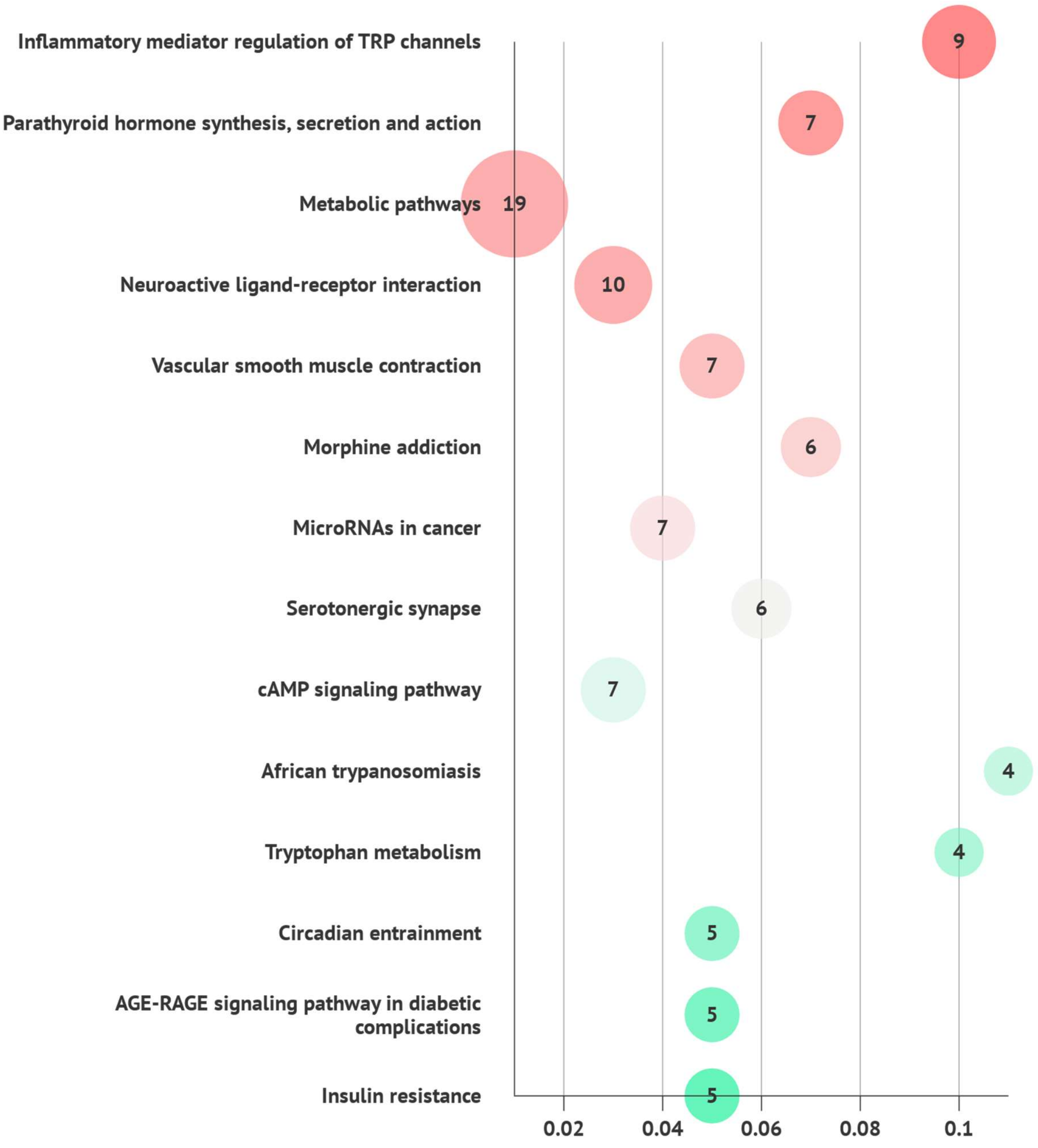

Figure S2. Pathway enrichment results based on STRING Enrichment. X-axis showed the value of rich factor, the red color showed lower Q-value, and the number inside the bubble showed gene count.

Table S4. KEGG pathway enrichment analysis by STRING Enrichment.

o..	Pathway	Rich Factor	Gene Count	Q-Value	Genes
1	Inflammatory mediator regulation of TRP channels	0.1	9	1.54E-08	MAPK14|TRPA1|PRKCG|PRKCE|PRKCH|PRKCD|PRKCA|HTR2A|ENSP00000459962
2	Parathyroid hormone synthesis, secretion and action	0.07	7	1.15E-05	MMP13|PRKCG|EGFR|PDE4B|PDE4D|PDE4A|PRKCA
3	Metabolic pathways	0.01	19	1.31E-05	DAO|KYNU|SQLE|CA2|HMGCR|CYP2A6|PLA2G1B|PLB1|PDE4B|MAOA|PDE4D|PTGS1|HSD11B1|DHCR24|MAOB|PDE4A|VKORC1|CA1|IDO1
4	Neuroactive ligand-receptor interaction	0.03	10	1.31E-05	MTNR1B|MTNR1A|F2|ADORA2A|ADORA1|CNR1|TACR2|CNR2|HTR2A|ENSP00000459962
5	Vascular smooth muscle contraction	0.05	7	0.000024	PRKCG|PRKCE|PLA2G1B|PRKCH|ADORA2A|PRKCD|PRKCA
6	Morphine addiction	0.07	6	3.67E-05	PRKCG|PDE4B|PDE4D|ADORA1|PDE4A|PRKCA
7	MicroRNAs in cancer	0.04	7	5.64E-05	CDC25B|PRKCG|STAT3|EGFR|CDC25A|PRKCE|PRKCA
8	Serotonergic synapse	0.06	6	8.04E-05	PRKCG|MAOA|PTGS1|MAOB|PRKCA|HTR2A
9	cAMP signaling pathway	0.03	7	0.00023	GLI1|PDE4B|ADORA2A|PDE4D|ADORA1|PDE4A|PPARA
10	African trypanosomiasis	0.11	4	0.00029	PRKCG|FAS|PRKCA|IDO1
11	Tryptophan metabolism	0.1	4	0.00042	KYNU|MAOA|MAOB|IDO1
12	Circadian entrainment	0.05	5	0.00045	PER2|MTNR1B|PRKCG|MTNR1A|PRKCA
13	AGE-RAGE signaling pathway in diabetic complications	0.05	5	0.00056	MAPK14|STAT3|PRKCE|PRKCD|PRKCA
14	Insulin resistance	0.05	5	0.00077	STAT3|PRKCE|PRKCD|PPARA|NR1H3
15	Rap1 signaling pathway	0.03	6	0.001	MAPK14|PRKCG|EGFR|ADORA2A|CNR1|PRKCA
16	Calcium signaling pathway	0.03	6	0.001	PRKCG|EGFR|ADORA2A|TACR2|PRKCA|HTR2A
17	Sphingolipid signaling pathway	0.04	5	0.001	MAPK14|PRKCG|PRKCE|ADORA1|PRKCA
18	Pathways in cancer	0.02	9	0.001	GLI1|PRKCG|STAT3|EGFR|F2|FAS|AR|GLI2|PRKCA
19	Proteoglycans in cancer	0.03	6	0.001	MAPK14|PRKCG|STAT3|EGFR|FAS|PRKCA
20	Dopaminergic synapse	0.04	5	0.0012	MAPK14|PRKCG|MAOA|MAOB|PRKCA
21	Amphetamine addiction	0.06	4	0.0013	PRKCG|MAOA|MAOB|PRKCA
22	Human cytomegalovirus infection	0.03	6	0.0014	MAPK14|PRKCG|STAT3|EGFR|FAS|PRKCA

23	Non-small cell lung cancer	0.06	4	0.0014	PRKCG|STAT3|EGFR|PRKCA
24	Retrograde endocannabinoid signaling	0.03	5	0.0018	MAPK14|FAAH|PRKCG|CNR1|PRKCA
25	EGFR tyrosine kinase inhibitor resistance	0.05	4	0.002	PRKCG|STAT3|EGFR|PRKCA
26	Hepatitis C	0.03	5	0.0023	STAT3|EGFR|FAS|PPARA|NR1H3
27	Hepatitis B	0.03	5	0.0024	MAPK14|PRKCG|STAT3|FAS|PRKCA
28	Gap junction	0.05	4	0.0027	PRKCG|EGFR|PRKCA|HTR2A
29	Fc gamma R-mediated phagocytosis	0.04	4	0.0028	PRKCG|PRKCE|PRKCD|PRKCA
30	GnRH signaling pathway	0.04	4	0.0028	MAPK14|EGFR|PRKCD|PRKCA
31	Progesterone-mediated oocyte maturation	0.04	4	0.0032	MAPK14|CDC25B|PLK1|CDC25A
32	Glycine, serine and threonine metabolism	0.08	3	0.0033	DAO|MAOA|MAOB
33	Choline metabolism in cancer	0.04	4	0.0033	PRKCG|SLC5A7|EGFR|PRKCA
34	Pancreatic secretion	0.04	4	0.0034	PRKCG|CA2|PLA2G1B|PRKCA
35	MAPK signaling pathway	0.02	6	0.0037	MAPK14|CDC25B|PRKCG|EGFR|FAS|PRKCA
36	HIF-1 signaling pathway	0.04	4	0.0043	PRKCG|STAT3|EGFR|PRKCA
37	Cholinergic synapse	0.04	4	0.0048	PRKCG|SLC5A7|ACHE|PRKCA
38	Arginine and proline metabolism	0.06	3	0.0054	DAO|MAOA|MAOB
39	Growth hormone synthesis, secretion and action	0.03	4	0.0058	MAPK14|PRKCG|STAT3|PRKCA
40	FoxO signaling pathway	0.03	4	0.0074	MAPK14|STAT3|EGFR|PLK1
41	Relaxin signaling pathway	0.03	4	0.0074	MAPK14|MMP13|EGFR|PRKCA
42	VEGF signaling pathway	0.05	3	0.0079	MAPK14|PRKCG|PRKCA
43	Arachidonic acid metabolism	0.05	3	0.0093	PLA2G1B|PLB1|PTGS1
44	Drug metabolism - cytochrome P450	0.05	3	0.0104	CYP2A6|MAOA|MAOB
45	Phenylalanine metabolism	0.12	2	0.0129	MAOA|MAOB
46	Nitrogen metabolism	0.12	2	0.0129	CA2|CA1
47	Glioma	0.04	3	0.0135	PRKCG|EGFR|PRKCA
48	Gastric acid secretion	0.04	3	0.0137	PRKCG|CA2|PRKCA
49	Steroid biosynthesis	0.1	2	0.0159	SQLE|DHCR24

50	Histidine metabolism	0.1	2	0.017	MAOA|MAOB
51	ErbB signaling pathway	0.04	3	0.0184	PRKCG|EGFR|PRKCA
52	PD-L1 expression and PD-1 checkpoint pathway in cancer	0.03	3	0.0212	MAPK14|STAT3|EGFR
53	alpha-Linolenic acid metabolism	0.08	2	0.0221	PLA2G1B|PLB1
54	Aldosterone synthesis and secretion	0.03	3	0.0251	PRKCG|PRKCE|PRKCA
55	Glycerophospholipid metabolism	0.03	3	0.0261	ACHE|PLA2G1B|PLB1
56	Linoleic acid metabolism	0.07	2	0.0275	PLA2G1B|PLB1
57	Human immunodeficiency virus 1 infection	0.02	4	0.0279	MAPK14|PRKCG|FAS|PRKCA
58	Leukocyte transendothelial migration	0.03	3	0.0339	MAPK14|PRKCG|PRKCA
59	Shigellosis	0.02	4	0.0339	MAPK14|EGFR|PRKCE|PRKCD
60	Tyrosine metabolism	0.06	2	0.0363	MAOA|MAOB
61	Neurotrophin signaling pathway	0.03	3	0.0364	MAPK14|PSEN2|PRKCD
62	Ras signaling pathway	0.02	4	0.0365	PRKCG|EGFR|PLA2G1B|PRKCA
63	Aldosterone-regulated sodium reabsorption	0.05	2	0.0383	PRKCG|PRKCA
64	Cell cycle	0.03	3	0.0398	CDC25B|PLK1|CDC25A
65	Oocyte meiosis	0.03	3	0.0398	MAPK14|PLK1|AR
66	Platelet activation	0.02	3	0.0398	MAPK14|F2|PTGS1
67	Natural killer cell mediated cytotoxicity	0.02	3	0.0398	PRKCG|FAS|PRKCA
68	Parkinson disease	0.02	4	0.0408	LRRK2|ADORA2A|MAOA|MAOB
69	Purine metabolism	0.02	3	0.0429	PDE4B|PDE4D|PDE4A

Table S5. Grid box used for molecular docking.

No.	Protein	Center			Size		
		X	Y	Z	X	Y	Z
1	EGFR (7B85)	-0.767	-50.831	19.3676	13.6225	17.623	16.623
2	MAPK14 (6HWU)	-4.272	-2.402	-24.287	8.5	17	18
3	PPARA (6KXY)	14.4548	-12.293	-30.833	23.5	19.229	19.989

Table S6. Attracting Cavities (AC) docking scores.

No	Protein	Compound	AC Score (kcal/mol)
1	EGFR (7B85)	R28 (Control)	-11.79183
		7-Hydroxycoumarin	0.188962
		Oxypeucedanin	254.700437
2	MAPK14 (6HWU)	GE5 (Control)	-19.199325
		Citrusoside A	37.041177
3	PPARA (6KXY)	T06 (Control)	14.356196
		α-Thujene	207.771981
		α-Pinene	39.957755
		Limonene	-20.338006
		β-Myrcene	-14.829328
		α-Terpinolene	-13.791016
		α-Terpinene	-16.942914
		β-Caryophyllene	39.568651
		Copaene	37.527143
		δ-3-Carene	211.761295
		δ-Elemene	2.991802
		Sabinene	204.217884
		(*E*)-Ocimene	-18.467425
		Cosmene	-15.647447
		Neoalloocimene	-21.637745
		α-Myrcene	-20.871249
		Cadinene	-12.306715
		Phellandrene	-12.885496
		Germacrene D	1.527614

Table S7. Residue interaction with ligand: EGFR (A), MAPK14 (B), and PPARA (C) using AutoDock Vina.

A. EGFR (1: 7-Hydroxycoumarin, 2: Oxypeucedanin)

	Residues	Control	1	2
Hydrogen Bonds	LEU718	o		
	THR790	o	o	o
	MET793	o		o
	CYS797	o		
	ASP855	o		
	Total	5	1	2
Hydrophobic Interactions	LEU718	o		
	VAL726	o	o	o
	LYS728	o		
	LEU744	o		
	LEU792	o		o
	LEU844	o		o
	Total	6	1	3

B. MAPK14 (1: Citrusoside A)

	Residues	Control	1
Hydrogen Bonds	ARG49	o	
	GLY170	o	o
	Total	2	1
Hydrophobic Interactions	GLY33	o	
	VAL38	o	o
	ALA51	o	o
	PHE169	o	o
	ALA40	o	
	LEU108	o	
	ALA34	o	
	LYS53	o	o
	Total	8	4

C. PPARA (1: α-Thujene, 2: α-Pinene, 3: Limonene, 4: β-Myrcene, 5: α-Terpinolene, 6: α-Terpinene, 7: β-Caryophyllene, 8: Copaene, 9: δ-3-Carene, 10: δ-Elemene, 11: Sabinene, 12: (*E*)-Ocimene, 13: Cosmene, 14: Neoalloocimene, 15: α-Myrcene, 16: Cadinene, 17: Phellandrene, 18: Germacrene D)

	**Residues**	**Control**	**1**	**2**	**3**	**4**	**5**	**6**	**7**	**8**	**9**	**10**	**11**	**12**	**13**	**14**	**15**	**16**	**17**	**18**
Hydrogen Bonds	TYR314	○																		
	TYR464	○																		
	Total	2	0	0	0	0	0	0	0	0	0	0	0	0	0	0	0	0	0	0
Hydrophobic Interactions	PHE273	○	○	○	○	○	○	○			○	○	○	○	○	○	○	○		○
	CYS276	○		○	○	○	○					○	○	○	○	○	○	○	○	○
	ILE354	○	○	○	○	○	○				○	○	○	○	○	○	○	○	○	○
	MET355	○										○		○		○				○
	VAL444	○	○	○		○	○	○			○	○	○	○	○	○	○	○		○
	ILE447	○	○	○		○	○	○			○	○	○		○	○	○			○
	LEU456	○	○	○		○	○	○			○		○		○	○	○			
	Total	7	5	6	3	6	6	4	0	0	5	6	6	5	6	7	6	4	2	6

Table S8. Residue interaction with ligand: EGFR (A), MAPK14 (B), and PPARA (C) using Attracting Cavities 2.0.

A. EGFR (1: 7-Hydroxycoumarin, 2: Oxypeucedanin)

	Residues	Control	1	2
Hydrogen Bonds	PRO794	o		
	GLU762	o	o	o
	ARG841	o		
	ASN842	o		
	CYS797	o		
	ASP800	o		
	THR854	o		
	Total	7	1	1
Hydrophobic Interactions	ALA743	o	o	o
	LEU844	o	o	o
	LEU718	o		
	VAL726	o	o	o
	PHE723	o		o
	CYS797	o		
	Total	6	3	4

B. MAPK14 (1: Citrusoside A)

	Residues	Control	1
Hydrogen Bonds	LYS53	o	o
	MET109	o	
	GLY33	o	
	THR106	o	
	HSD107	o	
	Total	5	1
Hydrophobic Interactions	VAL38	o	o
	PHE169	o	o
	LEU108	o	o
	ALA51	o	o
	MET109	o	
	LEU171	o	
	LYS53	o	o
	ALA34	o	
	Total	8	5

C. PPARA (1: α-Thujene, 2: α-Pinene, 3: Limonene, 4: β-Myrcene, 5: α-Terpinolene, 6: α-Terpinene, 7: β-Caryophyllene, 8: Copaene, 9: δ-3-Carene, 10: δ-Elemene, 11: Sabinene, 12: (*E*)-Ocimene, 13: Cosmene, 14: Neoalloocimene, 15: α-Myrcene, 16: Cadinene, 17: Phellandrene, 18: Germacrene D)

	Residues	Control	1	2	3	4	5	6	7	8	9	10	11	12	13	14	15	16	17	18
Hydrogen Bonds	TYR314	○																		
	Total	1	0	0	0	0	0	0	0	0	0	0	0	0	0	0	0	0	0	0
Hydrophobic Interactions	CYS276	○	○		○	○	○	○			○	○		○	○	○	○	○	○	
	LEU321	○		○			○	○												
	MET355	○				○	○	○				○		○	○	○	○	○	○	
	VAL444	○				○								○						
	ILE447	○																		
	PHE273	○				○						○			○	○	○	○		
	ILE354	○				○						○		○	○	○	○	○	○	
	ILE272	○	○		○	○					○			○	○	○	○			
	Total	8	2	1	2	6	3	3	0	0	2	4	0	5	5	5	5	4	3	0

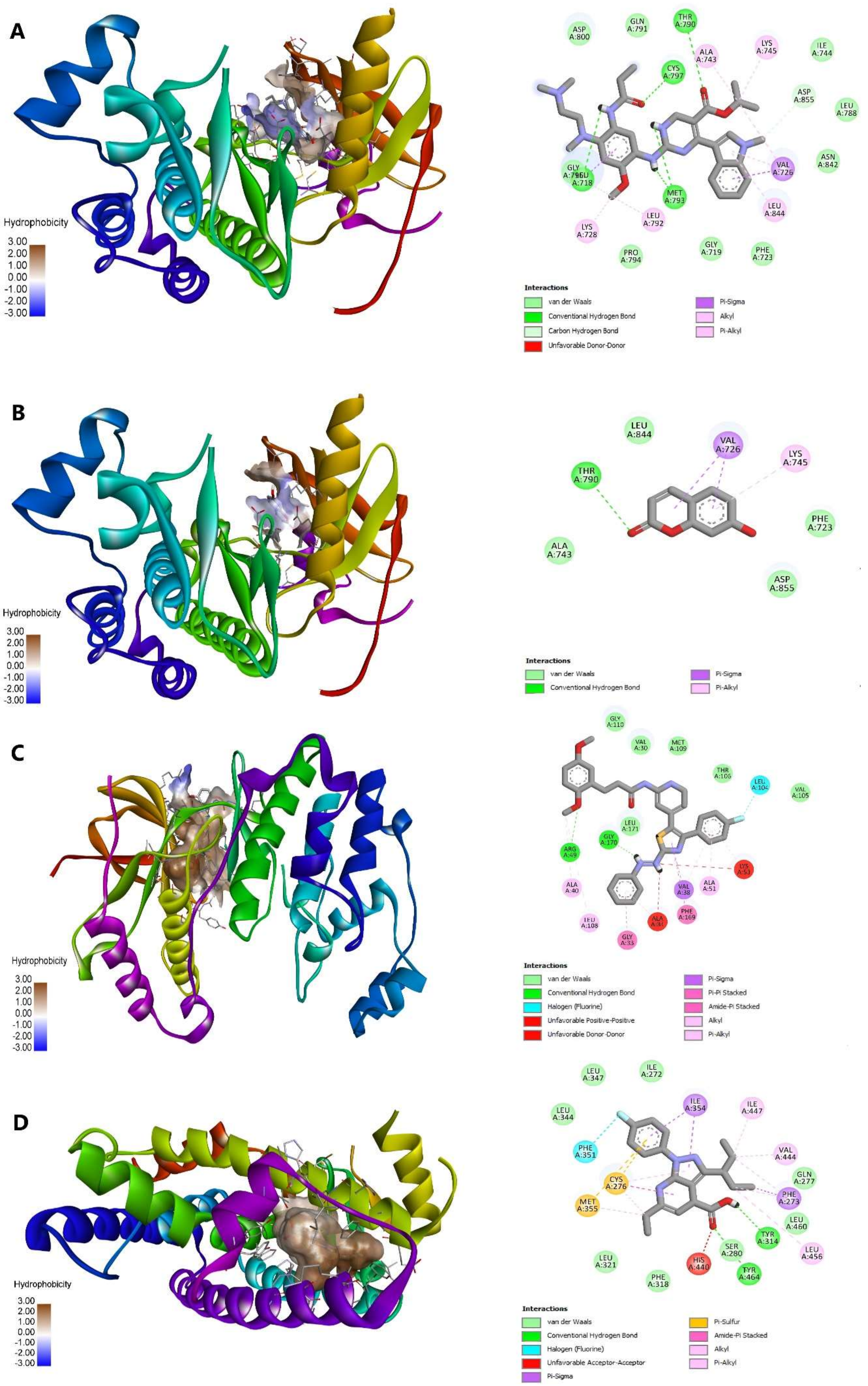
A
Hydrophobicity
3.00
2.00
1.00
0.00
-1.00
-2.00
-3.00
ASP A:800
GLN A:791
THR A:790
CYS A:797
ALA A:743
LYS A:745
ILE A:744
ASP A:855
LEU A:788
VAL A:726
ASN A:842
MET A:793
LEU A:792
LYS A:728
LEU A:844
PRO A:794
GLY A:719
PHE A:723
Interactions
van der Waals
Conventional Hydrogen Bond
Carbon Hydrogen Bond
Unfavorable Donor-Donor
Pi-Sigma
Alkyl
Pi-Alkyl
B
Hydrophobicity
3.00
2.00
1.00
0.00
-1.00
-2.00
-3.00
LEU A:844
VAL A:726
LYS A:745
THR A:790
PHE A:723
ALA A:743
ASP A:855
Interactions
van der Waals
Conventional Hydrogen Bond
Pi-Sigma
Pi-Alkyl
C
Hydrophobicity
3.00
2.00
1.00
0.00
-1.00
-2.00
-3.00
Interactions
van der Waals
Conventional Hydrogen Bond
Halogen (Fluorine)
Unfavorable Positive-Positive
Unfavorable Donor-Donor
Pi-Sigma
Pi-Pi Stacked
Amide-Pi Stacked
Alkyl
Pi-Alkyl
D
Hydrophobicity
3.00
2.00
1.00
0.00
-1.00
-2.00
-3.00
LEU A:347
ILE A:272
LEU A:344
ILE A:354
ILE A:447
PHE A:351
VAL A:444
GLN A:277
CYS A:276
PHE A:273
MET A:355
LEU A:460
TYR A:314
LEU A:456
SER A:280
HIS A:440
TYR A:464
LEU A:321
PHE A:318
Interactions
van der Waals
Conventional Hydrogen Bond
Halogen (Fluorine)
Unfavorable Acceptor-Acceptor
Pi-Sigma
Pi-Sulfur
Amide-Pi Stacked
Alkyl
Pi-Alkyl

E

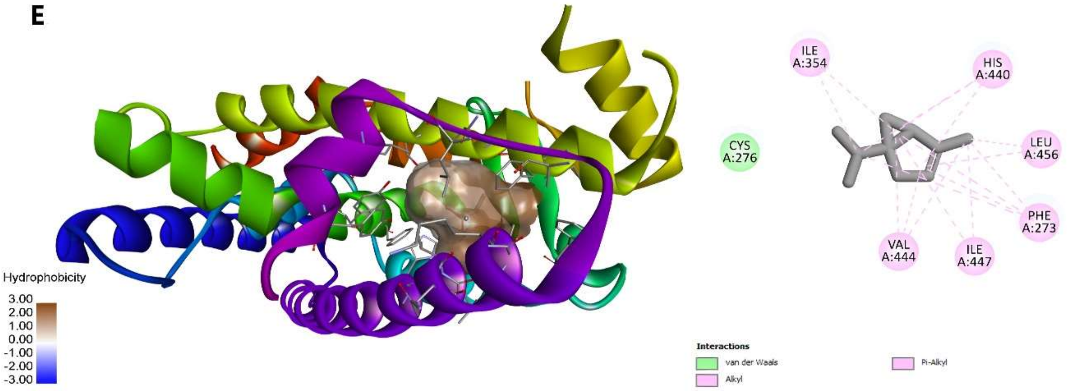
Hydrophobicity
3.00
2.00
1.00
0.00
-1.00
-2.00
-3.00
CYS A:276
ILE A:354
HIS A:440
LEU A:456
PHE A:273
VAL A:444
ILE A:447
Interactions
van der Waals
Alkyl
Pi-Alkyl

F

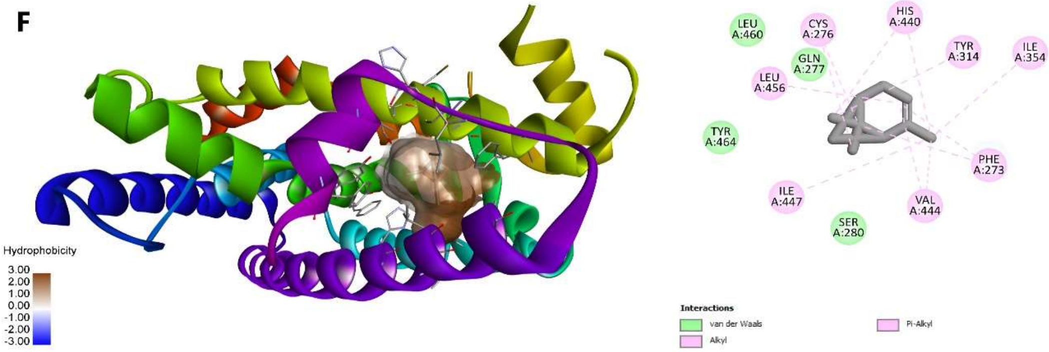
Hydrophobicity
3.00
2.00
1.00
0.00
-1.00
-2.00
-3.00
LEU A:460
CYS A:276
HIS A:440
TYR A:314
ILE A:354
GLN A:277
LEU A:456
TYR A:464
PHE A:273
ILE A:447
VAL A:444
SER A:280
Interactions
van der Waals
Alkyl
Pi-Alkyl

G

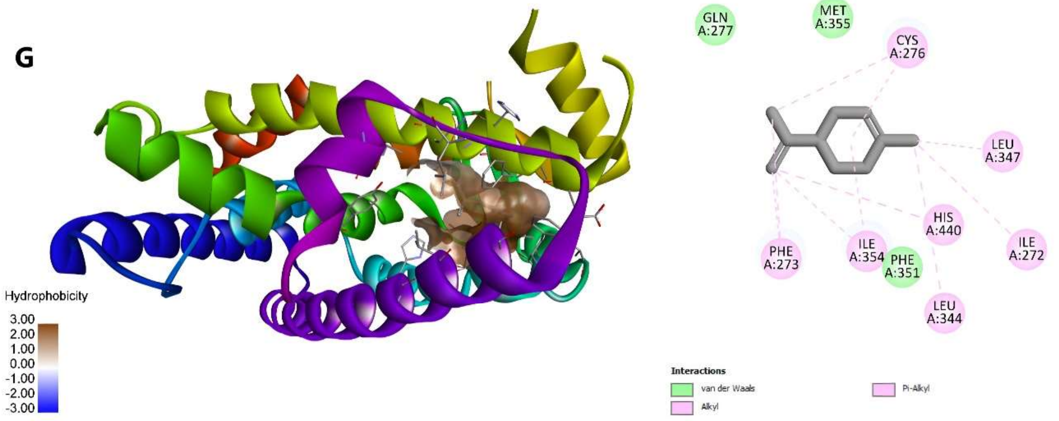
Hydrophobicity
3.00
2.00
1.00
0.00
-1.00
-2.00
-3.00
GLN A:277
MET A:355
CYS A:276
LEU A:347
HIS A:440
ILE A:272
PHE A:273
ILE A:354
PHE A:351
LEU A:344
Interactions
van der Waals
Alkyl
Pi-Alkyl

H

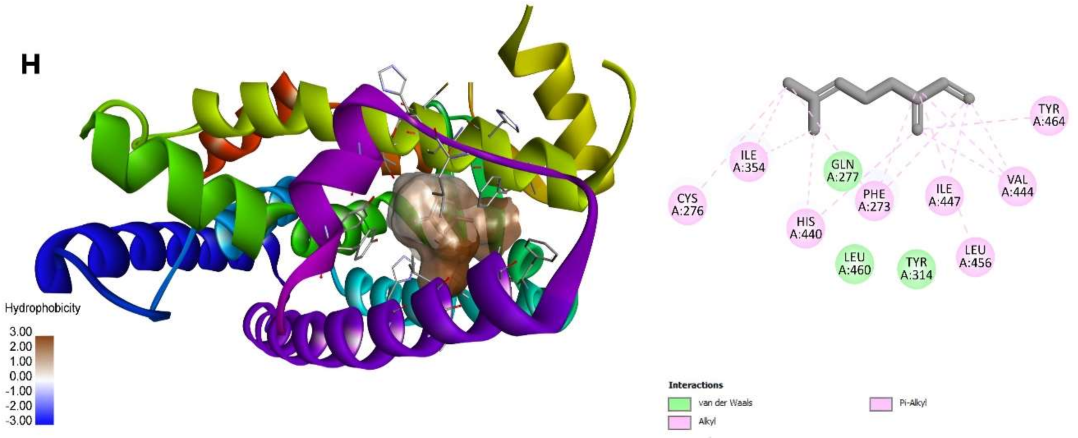
Hydrophobicity
3.00
2.00
1.00
0.00
-1.00
-2.00
-3.00
TYR A:464
ILE A:354
GLN A:277
VAL A:444
CYS A:276
PHE A:273
ILE A:447
HIS A:440
LEU A:460
TYR A:314
LEU A:456
Interactions
van der Waals
Alkyl
Pi-Alkyl

I

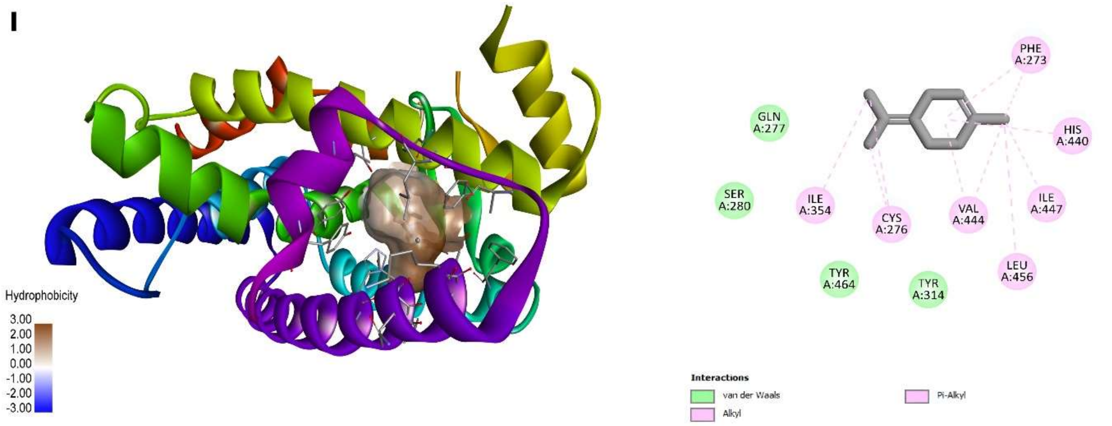
Hydrophobicity
3.00
2.00
1.00
0.00
-1.00
-2.00
-3.00
PHE A:273
GLN A:277
HIS A:440
SER A:280
ILE A:354
CYS A:276
VAL A:444
ILE A:447
TYR A:464
TYR A:314
LEU A:456
Interactions
van der Waals
Alkyl
Pi-Alkyl

J

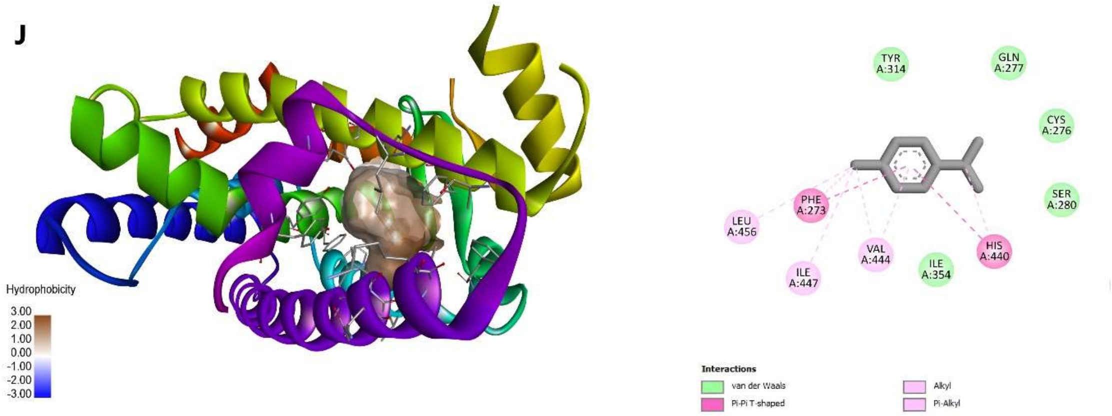
Hydrophobicity
3.00
2.00
1.00
0.00
-1.00
-2.00
-3.00
TYR A:314
GLN A:277
CYS A:276
PHE A:273
SER A:280
LEU A:456
VAL A:444
HIS A:440
ILE A:354
ILE A:447
Interactions
van der Waals
Pi-Pi T-shaped
Alkyl
Pi-Alkyl

K

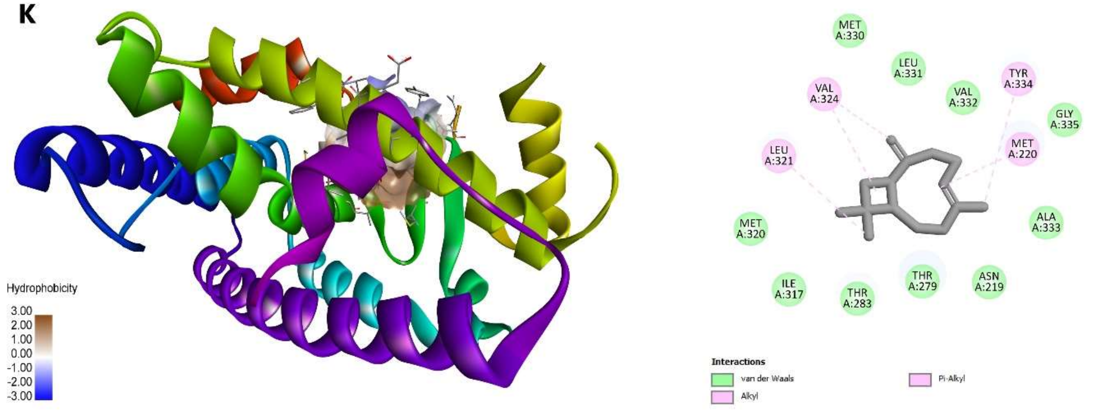
Hydrophobicity
3.00
2.00
1.00
0.00
-1.00
-2.00
-3.00
MET A:330
LEU A:331
VAL A:324
VAL A:332
TYR A:334
GLY A:335
LEU A:321
MET A:220
MET A:320
ALA A:333
ILE A:317
THR A:283
THR A:279
ASN A:219
Interactions
van der Waals
Alkyl
Pi-Alkyl

L

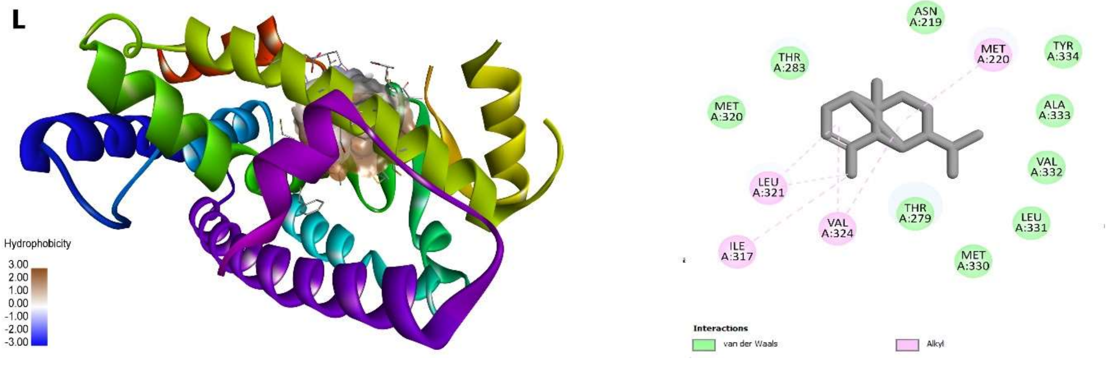
Hydrophobicity
3.00
2.00
1.00
0.00
-1.00
-2.00
-3.00
ASN A:219
MET A:220
TYR A:334
THR A:283
MET A:320
ALA A:333
VAL A:332
LEU A:321
THR A:279
VAL A:324
LEU A:331
ILE A:317
MET A:330
Interactions
van der Waals
Alkyl

M

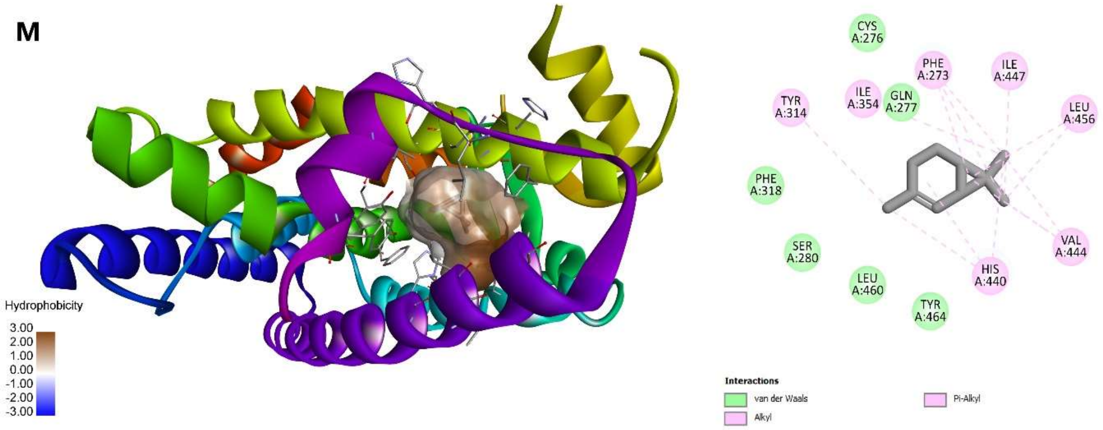
Hydrophobicity
3.00
2.00
1.00
0.00
-1.00
-2.00
-3.00
CYS A:276
PHE A:273
ILE A:447
TYR A:314
ILE A:354
GLN A:277
LEU A:456
PHE A:318
SER A:280
VAL A:444
HIS A:440
LEU A:460
TYR A:464
Interactions
van der Waals
Alkyl
Pi-Alkyl

N

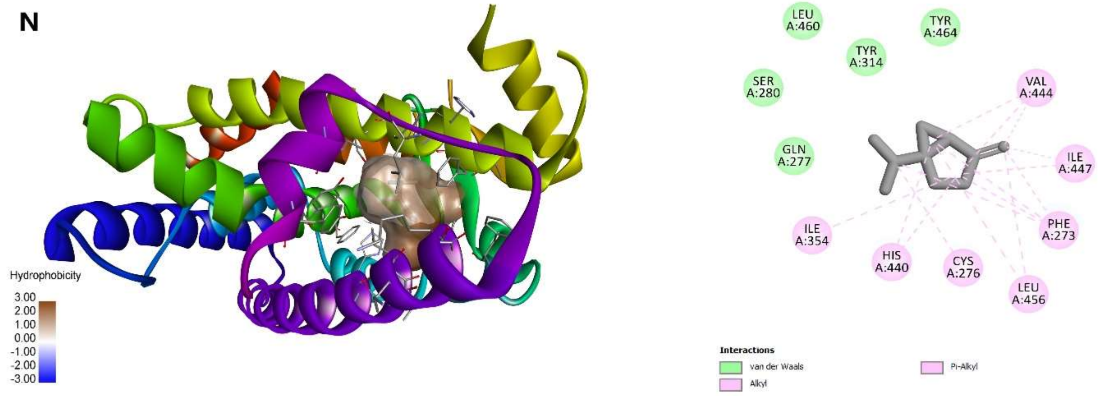
Hydrophobicity
3.00
2.00
1.00
0.00
-1.00
-2.00
-3.00
LEU A:460
TYR A:464
TYR A:314
SER A:280
VAL A:444
GLN A:277
ILE A:447
PHE A:273
ILE A:354
HIS A:440
CYS A:276
LEU A:456
Interactions
van der Waals
Alkyl
Pi-Alkyl

O

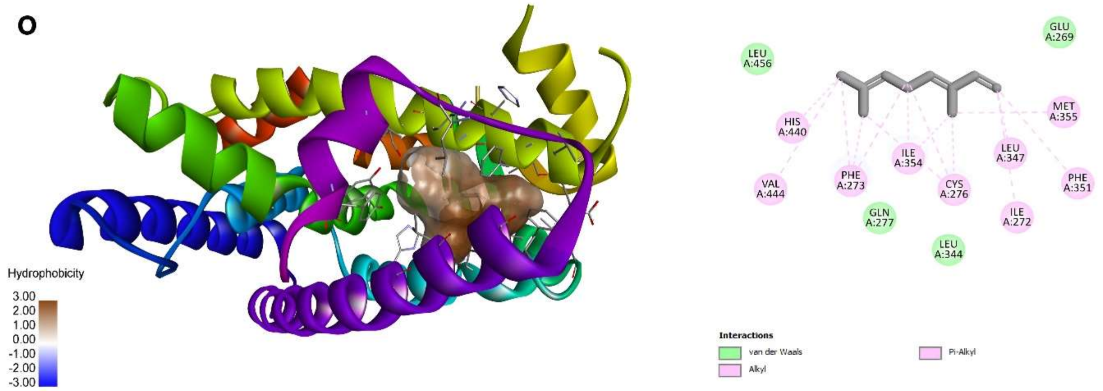
Hydrophobicity
3.00
2.00
1.00
0.00
-1.00
-2.00
-3.00
GLU A:269
LEU A:456
MET A:355
HIS A:440
LEU A:347
ILE A:354
PHE A:351
VAL A:444
PHE A:273
CYS A:276
ILE A:272
GLN A:277
LEU A:344
Interactions
van der Waals
Alkyl
Pi-Alkyl

P

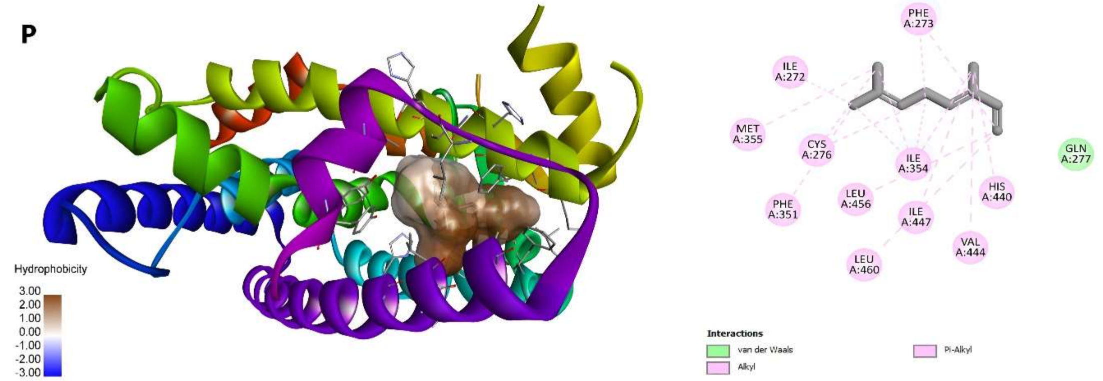
Hydrophobicity
3.00
2.00
1.00
0.00
-1.00
-2.00
-3.00
PHE A:273
ILE A:272
MET A:355
CYS A:276
GLN A:277
ILE A:354
HIS A:440
PHE A:351
LEU A:456
ILE A:447
VAL A:444
LEU A:460
Interactions
van der Waals
Alkyl
Pi-Alkyl

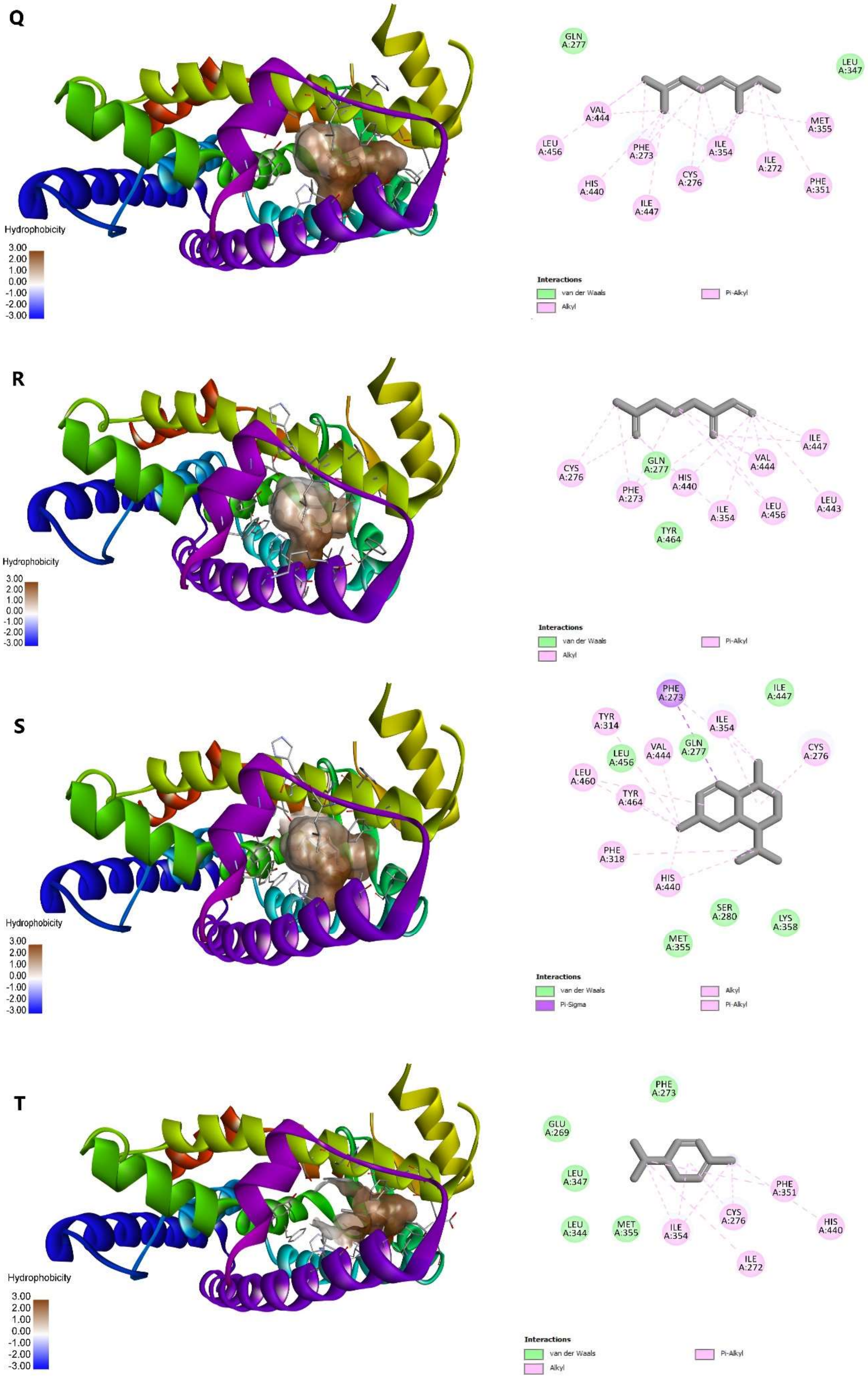

Q
Hydrophobicity
3.00
2.00
1.00
0.00
-1.00
-2.00
-3.00
GLN A:277
LEU A:347
VAL A:444
MET A:355
LEU A:456
PHE A:273
ILE A:354
ILE A:272
CYS A:276
HIS A:440
PHE A:351
ILE A:447
Interactions
van der Waals
Pi-Alkyl
Alkyl
R
Hydrophobicity
3.00
2.00
1.00
0.00
-1.00
-2.00
-3.00
ILE A:447
CYS A:276
GLN A:277
VAL A:444
HIS A:440
PHE A:273
ILE A:354
LEU A:456
LEU A:443
TYR A:464
Interactions
van der Waals
Pi-Alkyl
Alkyl
S
Hydrophobicity
3.00
2.00
1.00
0.00
-1.00
-2.00
-3.00
PHE A:273
ILE A:447
TYR A:314
ILE A:354
LEU A:456
VAL A:444
GLN A:277
CYS A:276
LEU A:460
TYR A:464
PHE A:318
HIS A:440
SER A:280
LYS A:358
MET A:355
Interactions
van der Waals
Alkyl
Pi-Sigma
Pi-Alkyl
T
Hydrophobicity
3.00
2.00
1.00
0.00
-1.00
-2.00
-3.00
PHE A:273
GLU A:269
LEU A:347
PHE A:351
CYS A:276
HIS A:440
LEU A:344
MET A:355
ILE A:354
ILE A:272
Interactions
van der Waals
Pi-Alkyl
Alkyl

Figure S3. AutoDock Vina docking visualization using Discovery Studio: EGFR—control (A), EGFR—7-hydroxycoumarin (B), MAPK14—control (C), PPARA—control (D), PPARA—α-thujene (E), PPARA—α-pinene (F), PPARA—limonene (G), PPARA—β-myrcene (H), PPARA—α-terpinolene (I), PPARA—α-terpinene (J), PPARA—β-caryophyllene (K), PPARA—copaene (L), PPARA—δ-3-carene (M), PPARA—sabinene (N), PPARA—(*E*)-ocimene (O), PPARA—cosmene (P), PPARA—neoalloocimene (Q), PPARA—α-myrcene (R), PPARA—cadinene (S), and PPARA—phellandrene (T). The visualization on the left shows the protein structure and its hydrophobicity, meanwhile the right picture reveals the residue interaction of the complex.

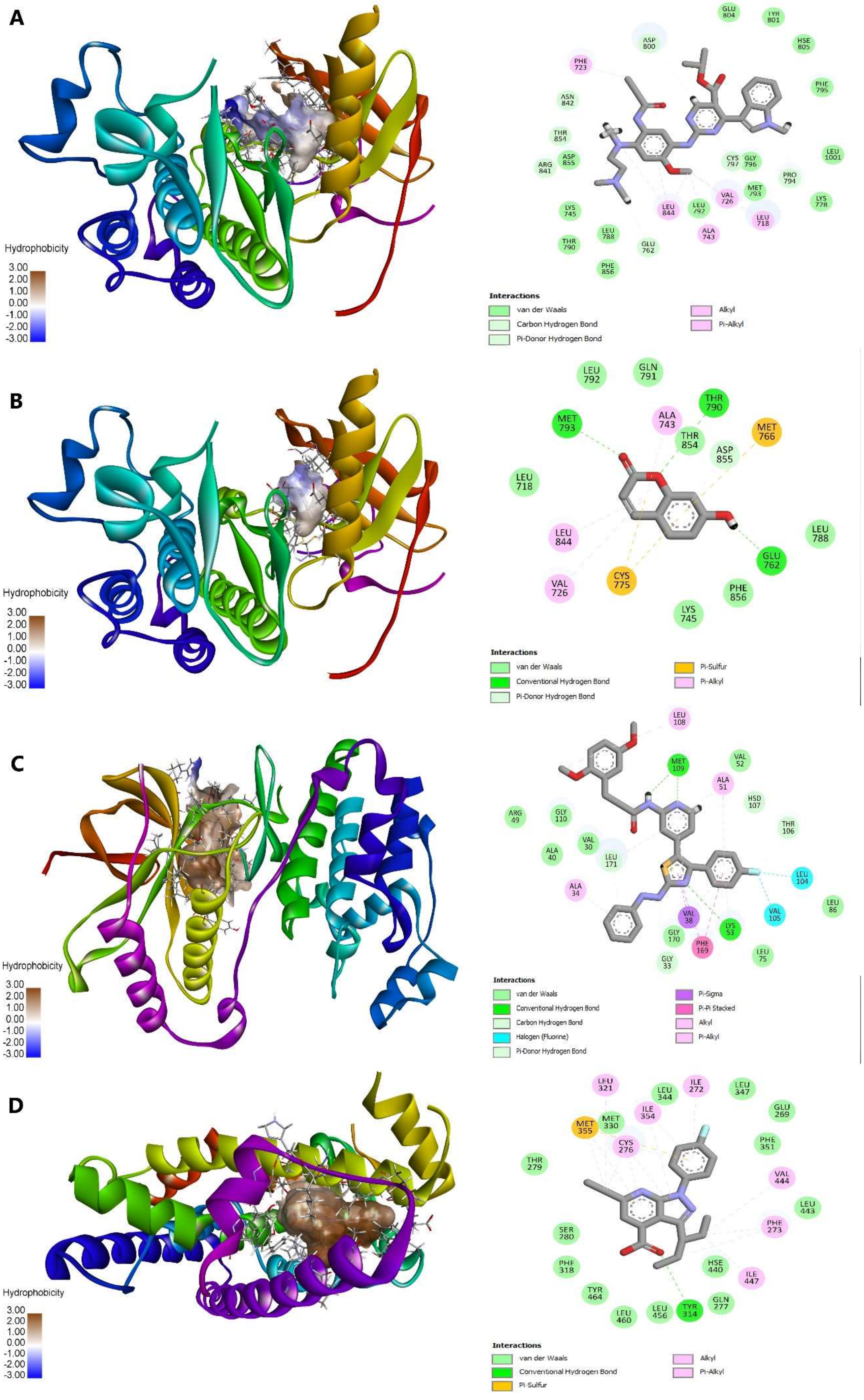
A
B
C
D
Hydrophobicity
3.00
2.00
1.00
0.00
-1.00
-2.00
-3.00
Interactions
van der Waals
Carbon Hydrogen Bond
Pi-Donor Hydrogen Bond
Alkyl
Pi-Alkyl
LEU 792
GLN 791
THR 790
MET 793
ALA 743
THR 854
ASP 855
MET 766
LEU 718
LEU 844
LEU 788
GLU 762
VAL 726
CYS 775
PHE 856
LYS 745
Conventional Hydrogen Bond
Pi-Sulfur
Halogen (Fluorine)
Pi-Sigma
Pi-Pi Stacked

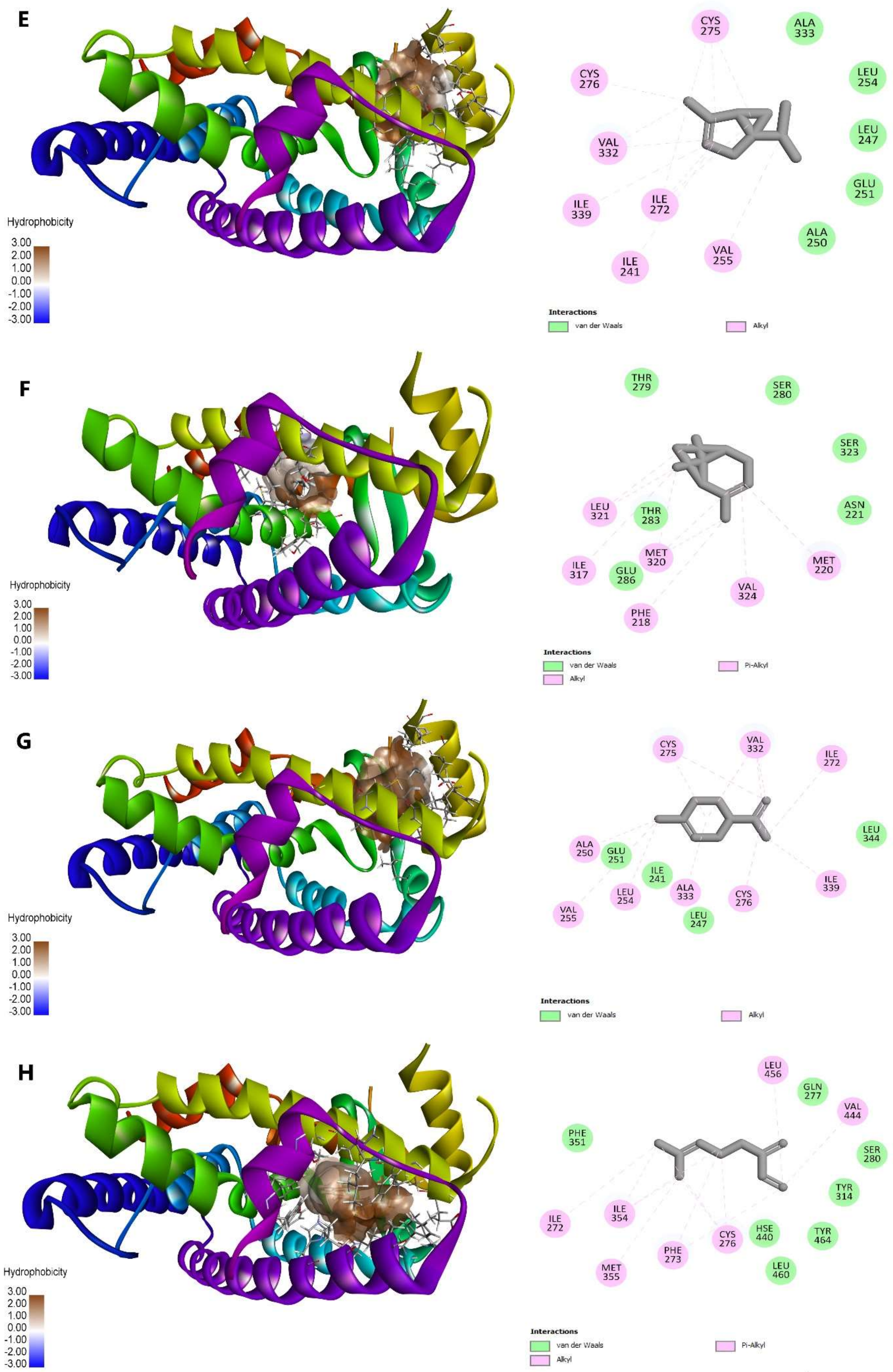
E
Hydrophobicity
3.00
2.00
1.00
0.00
-1.00
-2.00
-3.00
CYS 275
ALA 333
CYS 276
LEU 254
LEU 247
VAL 332
GLU 251
ILE 339
ILE 272
ALA 250
VAL 255
ILE 241
Interactions
van der Waals
Alkyl
F
Hydrophobicity
3.00
2.00
1.00
0.00
-1.00
-2.00
-3.00
THR 279
SER 280
SER 323
LEU 321
THR 283
ASN 221
ILE 317
MET 320
GLU 286
MET 220
VAL 324
PHE 218
Interactions
van der Waals
Pi-Alkyl
Alkyl
G
Hydrophobicity
3.00
2.00
1.00
0.00
-1.00
-2.00
-3.00
CYS 275
VAL 332
ILE 272
LEU 344
ALA 250
GLU 251
ILE 241
ALA 333
LEU 254
CYS 276
ILE 339
VAL 255
LEU 247
Interactions
van der Waals
Alkyl
H
Hydrophobicity
3.00
2.00
1.00
0.00
-1.00
-2.00
-3.00
LEU 456
GLN 277
VAL 444
PHE 351
SER 280
TYR 314
ILE 272
ILE 354
HSE 440
TYR 464
CYS 276
PHE 273
MET 355
LEU 460
Interactions
van der Waals
Pi-Alkyl
Alkyl

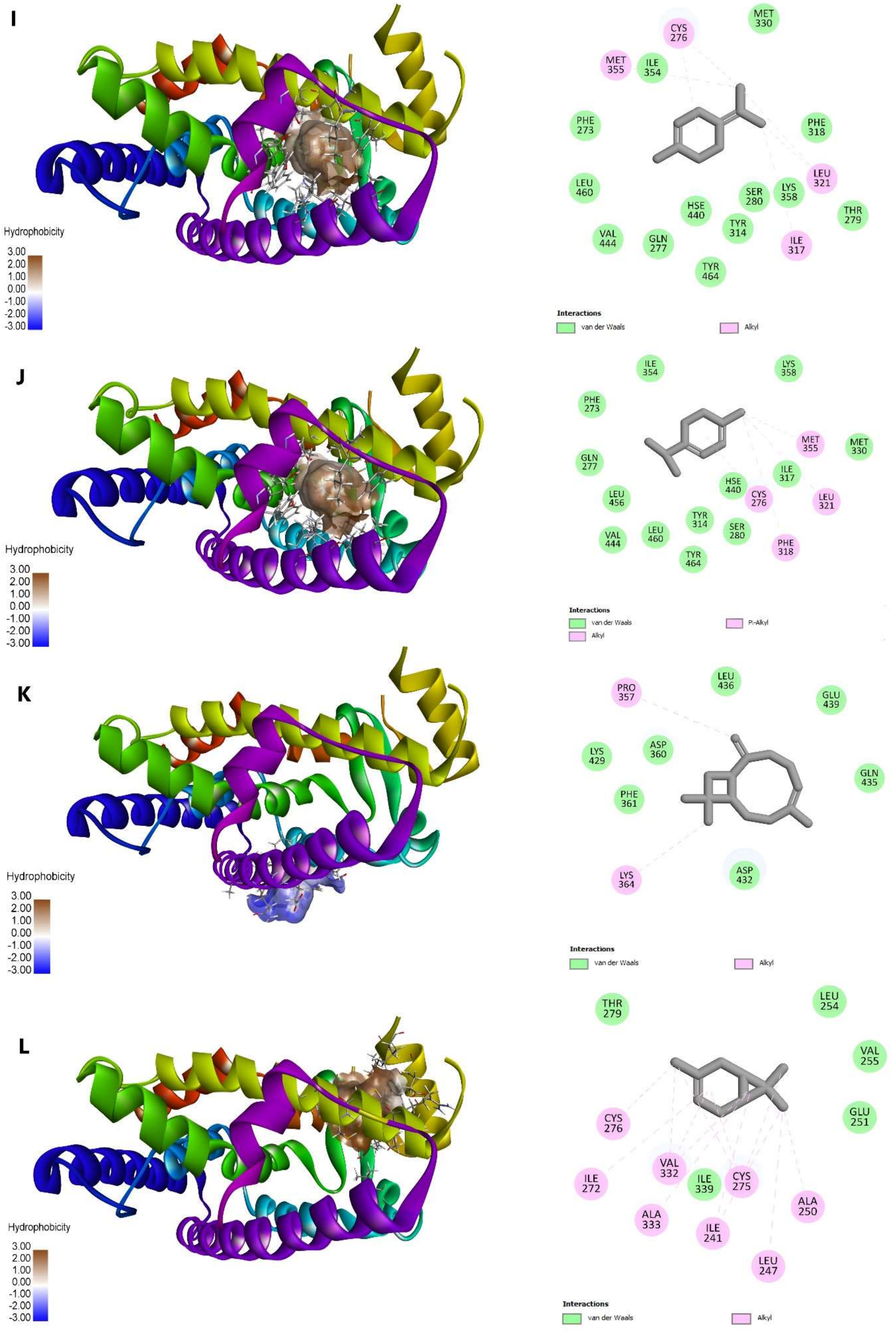
I
Hydrophobicity
3.00
2.00
1.00
0.00
-1.00
-2.00
-3.00
MET 330
CYS 276
MET 355
ILE 354
PHE 273
PHE 318
LEU 321
LEU 460
SER 280
LYS 358
HSE 440
THR 279
TYR 314
VAL 444
GLN 277
ILE 317
TYR 464
Interactions
van der Waals
Alkyl
J
Hydrophobicity
3.00
2.00
1.00
0.00
-1.00
-2.00
-3.00
ILE 354
LYS 358
PHE 273
MET 355
MET 330
GLN 277
ILE 317
HSE 440
LEU 456
CYS 276
LEU 321
TYR 314
VAL 444
LEU 460
SER 280
PHE 318
TYR 464
Interactions
van der Waals
Pi-Alkyl
Alkyl
K
Hydrophobicity
3.00
2.00
1.00
0.00
-1.00
-2.00
-3.00
PRO 357
LEU 436
GLU 439
LYS 429
ASP 360
GLN 435
PHE 361
LYS 364
ASP 432
Interactions
van der Waals
Alkyl
L
Hydrophobicity
3.00
2.00
1.00
0.00
-1.00
-2.00
-3.00
THR 279
LEU 254
VAL 255
GLU 251
CYS 276
VAL 332
ILE 272
ILE 339
CYS 275
ALA 250
ALA 333
ILE 241
LEU 247
Interactions
van der Waals
Alkyl

M

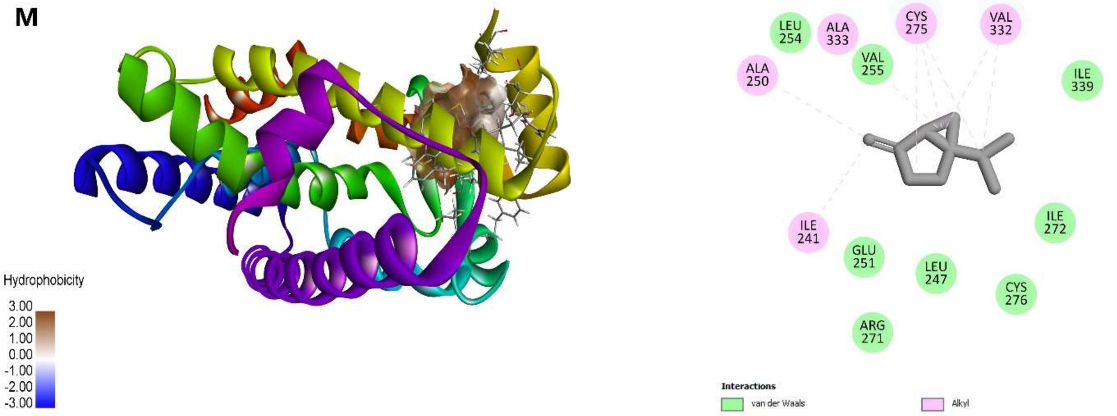
Hydrophobicity
3.00
2.00
1.00
0.00
-1.00
-2.00
-3.00
LEU 254
ALA 333
CYS 275
VAL 332
ALA 250
VAL 255
ILE 339
ILE 241
GLU 251
LEU 247
ILE 272
CYS 276
ARG 271
Interactions
van der Waals
Alkyl

N

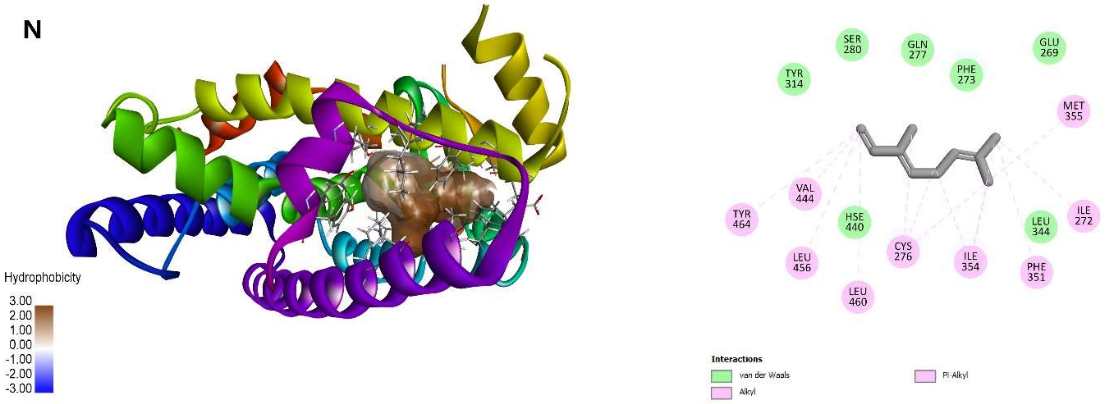
Hydrophobicity
3.00
2.00
1.00
0.00
-1.00
-2.00
-3.00
SER 280
GLN 277
GLU 269
TYR 314
PHE 273
MET 355
VAL 444
TYR 464
HSE 440
LEU 344
ILE 272
LEU 456
CYS 276
ILE 354
PHE 351
LEU 460
Interactions
van der Waals
Pi-Alkyl
Alkyl

O

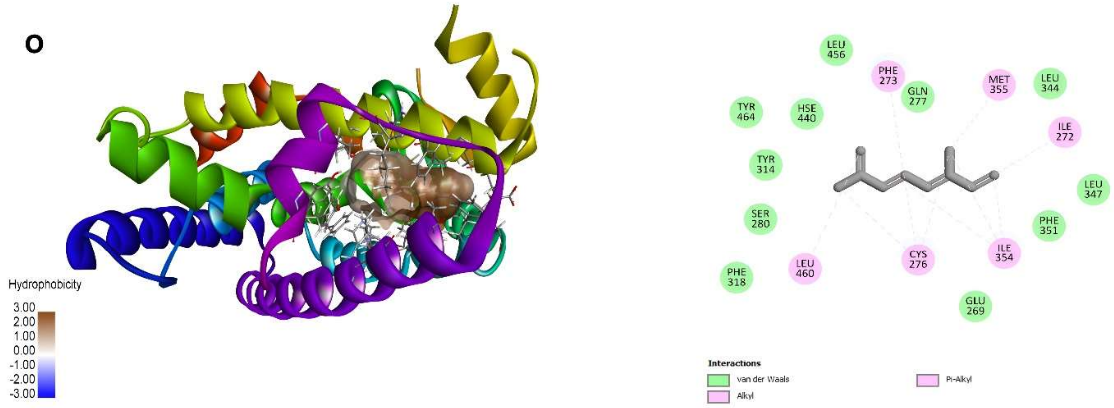
Hydrophobicity
3.00
2.00
1.00
0.00
-1.00
-2.00
-3.00
LEU 456
PHE 273
GLN 277
MET 355
LEU 344
TYR 464
HSE 440
ILE 272
TYR 314
LEU 347
SER 280
PHE 351
CYS 276
ILE 354
PHE 318
LEU 460
GLU 269
Interactions
van der Waals
Pi-Alkyl
Alkyl

P

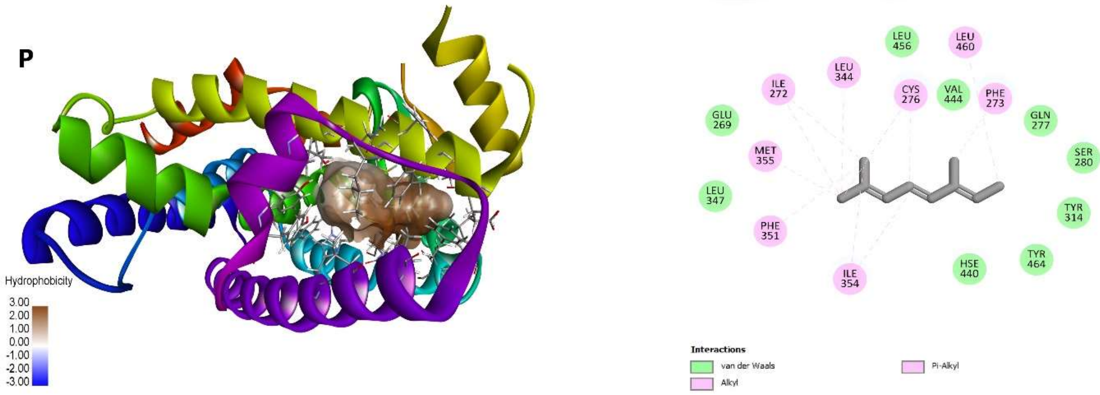
Hydrophobicity
3.00
2.00
1.00
0.00
-1.00
-2.00
-3.00
LEU 456
LEU 460
ILE 272
LEU 344
CYS 276
VAL 444
PHE 273
GLU 269
GLN 277
MET 355
SER 280
LEU 347
TYR 314
PHE 351
TYR 464
ILE 354
HSE 440
Interactions
van der Waals
Pi-Alkyl
Alkyl

Q

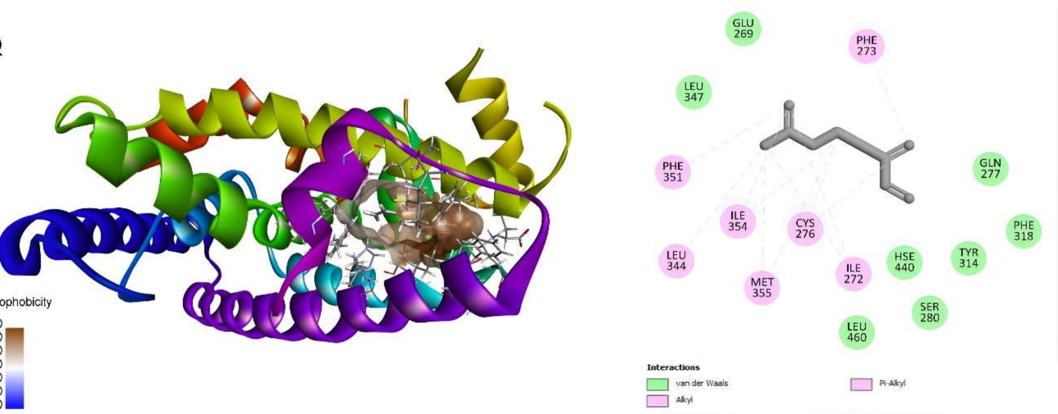
Hydrophobicity
3.00
2.00
1.00
0.00
-1.00
-2.00
-3.00
GLU 269
PHE 273
LEU 347
PHE 351
GLN 277
PHE 318
ILE 354
CYS 276
LEU 344
MET 355
ILE 272
HSE 440
TYR 314
SER 280
LEU 460
Interactions
van der Waals
Pi-Alkyl
Alkyl

R

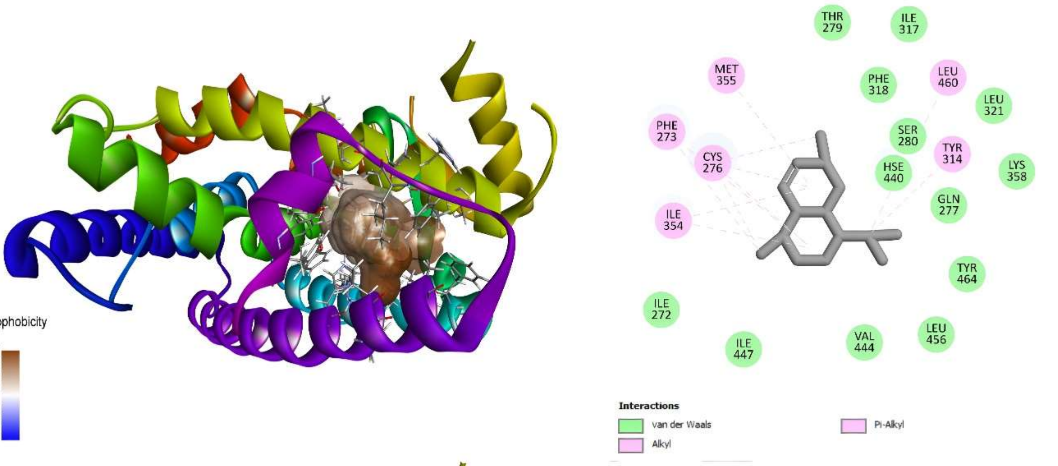
Hydrophobicity
3.00
2.00
1.00
0.00
-1.00
-2.00
-3.00
THR 279
ILE 317
MET 355
PHE 318
LEU 460
LEU 321
PHE 273
SER 280
CYS 276
TYR 314
LYS 358
HSE 440
GLN 277
ILE 354
TYR 464
ILE 272
ILE 447
VAL 444
LEU 456
Interactions
van der Waals
Pi-Alkyl
Alkyl

S

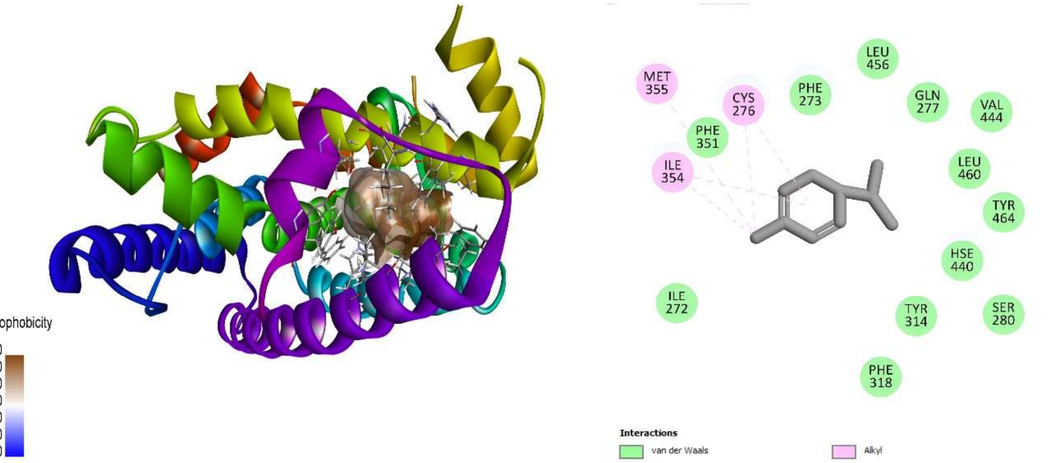
Hydrophobicity
3.00
2.00
1.00
0.00
-1.00
-2.00
-3.00
MET 355
LEU 456
CYS 276
PHE 273
GLN 277
VAL 444
PHE 351
ILE 354
LEU 460
TYR 464
HSE 440
ILE 272
TYR 314
SER 280
PHE 318
Interactions
van der Waals
Alkyl

T

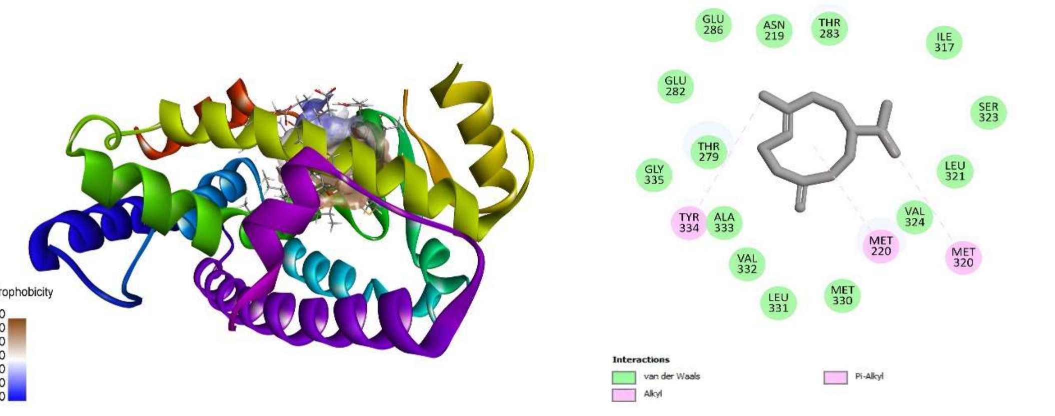
Hydrophobicity
3.00
2.00
1.00
0.00
-1.00
-2.00
-3.00
GLU 286
ASN 219
THR 283
ILE 317
GLU 282
SER 323
THR 279
GLY 335
LEU 321
TYR 334
ALA 333
VAL 324
MET 220
MET 320
VAL 332
LEU 331
MET 330
Interactions
van der Waals
Pi-Alkyl
Alkyl

Figure S4. Attracting Cavities 2.0 docking visualization using Discovery Studio: EGFR—control (A), EGFR—7-hydroxycoumarin (B), MAPK14—control (C), PPARA—control (D), PPARA—α-thujene (E), PPARA—α-pinene (F), PPARA—limonene (G), PPARA—β-myrcene (H), PPARA—α-terpinolene (I), PPARA—α-terpinene (J), PPARA—β-caryophyllene (K), PPARA—δ-3-carene (L), PPARA—sabinene (M), PPARA—(*E*)-ocimene (N), PPARA—cosmene (O), PPARA—neoalloocimene (P), PPARA—α-myrcene (Q), PPARA—cadinene (R), PPARA—phellandrene (S), and PPARA—germacrene D (T). The visualization on the left shows the protein structure and its hydrophobicity, meanwhile the right picture reveals the residue interaction of the complex.

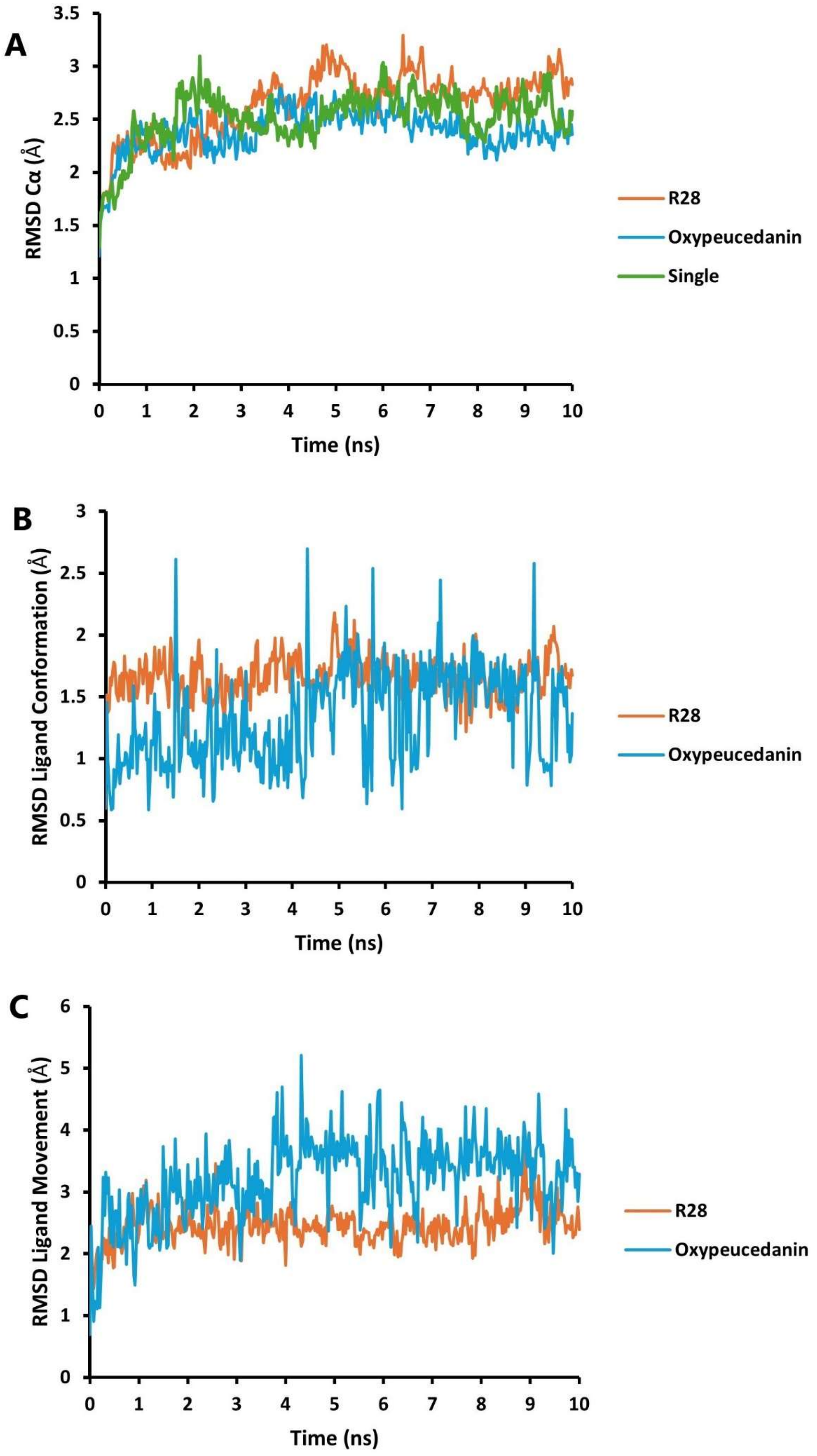

Figure S5. RMSD (Å): Cα (A), ligand conformation (B), and ligand movement (C) of EGFR single protein, EGFR—R28, and EGFR—oxypeucedanin complex.

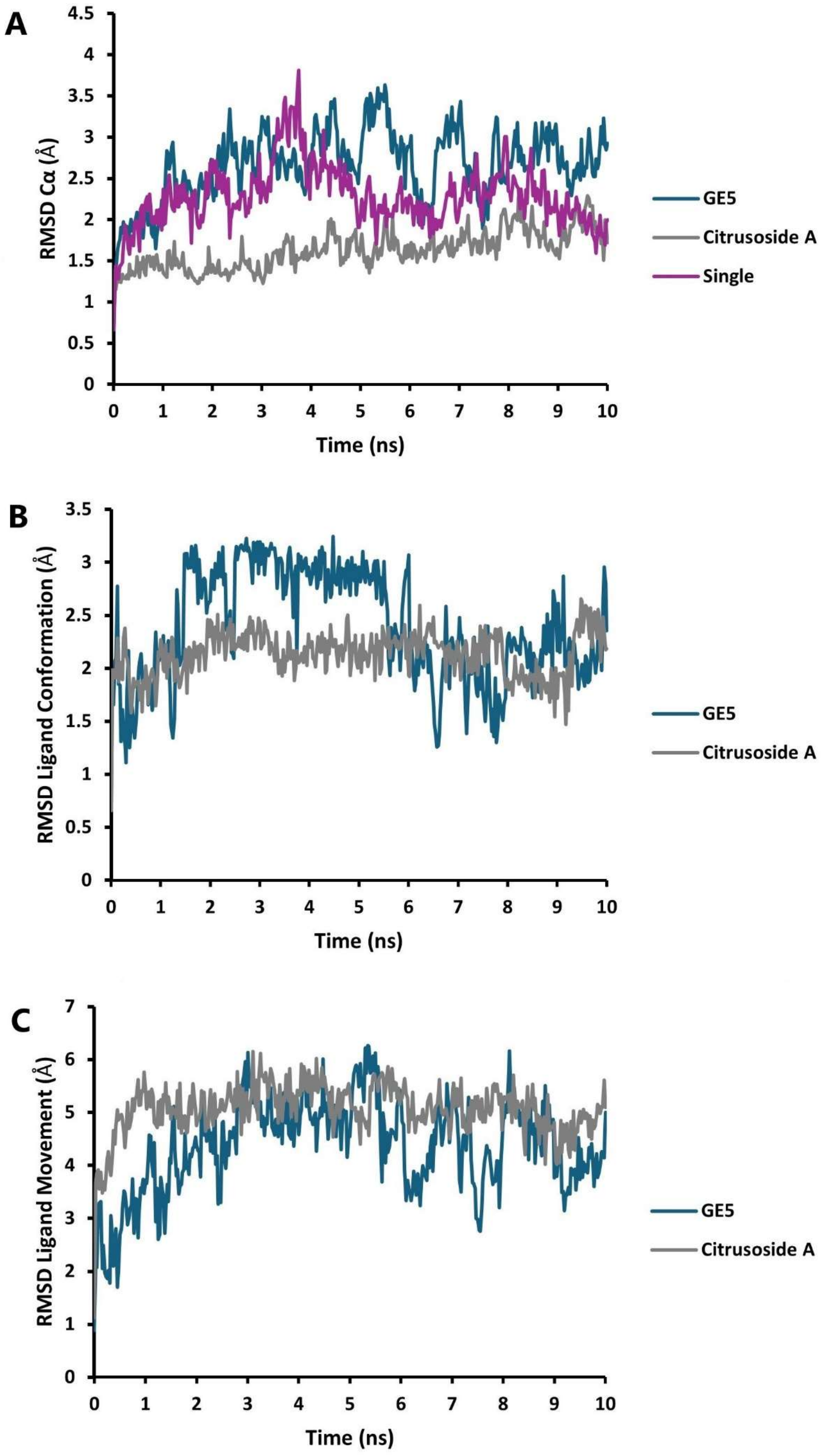

Figure S6. RMSD (Å): Cα (A), ligand conformation (B), and ligand movement (C) of MAPK14 single protein, MAPK14—GE5, and MAPK14—citrusoside A complex.
